# Recruitment of USP10 by GCS1 to deubiquitinate GRP78 promotes the progression of colorectal cancer via alleviating endoplasmic reticulum stress

**DOI:** 10.1186/s13046-024-03176-8

**Published:** 2024-09-13

**Authors:** Yang Chen, Hengyang Shen, Zhenling Wang, Changzhi Huang, Hongqiang Zhang, Yu Shao, Ying Tong, Lei Xu, Yunfei Lu, Zan Fu

**Affiliations:** 1 Department of General Surgery, The First Affiliated Hospital of Nanjing Medical University, 300 Guangzhou Road, Nanjing, Jiangsu 210009 P.R. China; 2 The First School of Clinical Medicine, Nanjing Medical University, Nanjing, Jiangsu China

**Keywords:** Colorectal cancer, GCS1, Endoplasmic reticulum stress, GRP78, Deubiquitination

## Abstract

**Background:**

Long-term accumulation of misfolded proteins leads to endoplasmic reticulum (ER) stress in colorectal cancer (CRC). However, the precise pathways controlling the decision between survival and apoptosis in CRC are unclear. Therefore, in this study, we investigated the function and molecular mechanism of glucosidase I (GCS1) in regulating ER stress in CRC.

**Methods:**

A public database was used to confirm the expression level of GCS1 in CRC and normal tissues. Clinical samples from our center were used to confirm the mRNA and protein expression levels of GCS1. Cell proliferation, migration, invasion, and apoptosis assays revealed the biological role of GCS1. Immunohistochemical techniques were used to evaluate the expression of key proteins in subcutaneous implanted tumors in nude mice, which provided further evidence for the biological function of GCS1 in promoting cancer in vivo. The results of coimmunoprecipitation-mass spectrometry analysis and immunofluorescence colocalization analysis the interaction between GCS1 and GRP78. In addition, the mechanism of action of USP10, GRP78, and GCS1 at the post- translational level was investigated. Finally, a tissue microarray was used to examine the connection between GCS1 and GRP78 expression and intracellular localization of these proteins using immunohistochemistry and immunofluorescence.

**Results:**

The experimental results revealed that GCS1 was substantially expressed in CRC, with higher expression indicating a worse prognosis. Thus, GCS1 can enhance the proliferation and metastasis while inhibiting the apoptosis of CRC cells both in vivo and in vitro. Mechanistically, GCS1 binds to GRP78, recruits USP10 for deubiquitination of GRP78 to promote its degradation, and decreases ER stress-mediated apoptosis, increasing CRC cell proliferation and metastasis.

**Conclusions:**

In summary, GCS1 stimulates CRC growth and migration and reduces ER stress-mediated apoptosis via USP10-mediated deubiquitination of GRP78. Our findings identify a possible therapeutic target for CRC.

**Supplementary Information:**

The online version contains supplementary material available at 10.1186/s13046-024-03176-8.

## Introduction

Colorectal cancer (CRC) is a prevalent malignant tumor, that ranks third in incidence and second in mortality among all cancers, and poses a serious threat to global health [1]. Although various treatment options, including endoscopic therapy, surgery, chemotherapy, immunotherapy, and molecular- targeted therapy, are available, the 5- year survival rate of CRC patients remains low [2–4]. Therefore, improving our understanding of the pathological molecular mechanisms of CRC and exploring new critical targets related to CRC progression and prognosis are of great practical importance for improving patient outcomes.

Accumulating studies indicate that the unfolded protein response (UPR) is closely related to the development of CRC [5]. The endoplasmic reticulum (ER), the primary organelle involved in the UPR, is crucial for protein synthesis, maturation, and transport [6, 7]. When ER homeostasis is disrupted, leading to excessive accumulation of unfolded or misfolded proteins, ER stress occurs, activating the UPR [8]. Typically, GRP78 binds to three key sensors, namely, PERK, IRE1, and ATF6, maintaining them in an inactive state. Under cellular stress conditions, GRP78 dissociates from these sensors, binds to unfolded proteins, and activates downstream signaling pathways to restore cellular homeostasis [9]. Under severe and irreparable ER stress conditions, apoptosis is induced via the PERK/eIF2α/ATF4/CHOP pathway [10–12].

By a preliminary multi-omics analysis by our research group [13], combined with clinical information from public databases and clinicopathological data from our center, glucosidase I (GCS1) was identified as a novel protein with prognostic value in CRC. GCS1, also known as MOGS, is an ER-anchored glucosidase involved primarily in glycoprotein processing [14]. Previous studies have shown that its expression is upregulated after peripheral nerve injury [15] and have identified elevated GCS1 expression in the brain capillaries of Alzheimer’s disease patients [16]. Another study confirmed that GCS1 alleviates ER stress as a binding protein of p32 [17]. However, whether GCS1 is involved in the regulation of ER stress in CRC and the specific mechanisms involved remain unclear, and further investigation is necessary.

GRP78, also known as HSPA5 or BiP, is a member of the 70 kDa heat shock protein (HSP70) family of molecular chaperones. Previous studies have confirmed the strong correlation between increased GRP78 expression and tumor aggressiveness and metastasis [18–20]. In addition, GRP78 is crucial for regulating the UPR induced by ER stress and plays a crucial role in tumor progression by inhibiting apoptosis [21–24]. For instance, in osteosarcoma, GRP78 inhibits apoptotic pathways via ubiquitination of CHOP [25]. In hepatocellular carcinoma, CD147 induces the UPR and ultimately inhibits apoptosis by increasing GRP78 transcription [26]. GRP78 also plays an essential role in CRC. For example, ATAD3A stabilizes GRP78 to inhibit ER stress, thereby endowing CRC with chemoresistance [27]. Klotho, a tumor suppressor in various malignancies, regulates the UPR through GRP78 to inhibit CRC progression [28]. These findings indicate that GRP78 is indispensable for tumor progression, especially in CRC.

In this study, we observed that elevated GCS1 expression in CRC tissues was positively correlated with tumor progression and indicated a poor prognosis in CRC patients. Additionally, the results of both in vitro and in vivo experiments demonstrated that GCS1 significantly promoted cell proliferation and metastasis while reducing ER stress-mediated apoptosis in CRC. Mechanistically, GCS1 was found to recruit the deubiquitinating enzyme (DUB) USP10 to remove lysine 48 (K48)-linked polyubiquitin chains from GRP78, preventing its degradation, which resulted in reduced CHOP expression during ER stress, and accelerated the malignant progression of CRC. In conclusion, our findings suggest that targeting the GCS1-USP10-GRP78 axis may be a potential strategy for the clinical treatment of CRC and prognostic prediction of CRC patient outcomes.

## Materials and methods

### Clinical patient samples and tissue microarray (TMA)

CRC samples and samples of the adjacent normal mucosa were collected from patients with CRC who underwent surgery at the First Affiliated Hospital of Nanjing Medical University between 2017 and 2020. The specimens were either placed in a -80 °C freezer or embedded in paraffin within 5 min of resection. Clinical samples from 80 patients were used for construction of a microarray of formalin-fixed, paraffin-embedded tissues by Servicebio (Wuhan, China). The First Affiliated Hospital of Nanjing Medical University’s Ethics Committee approved all experiments, in accordance with the principles of the Declaration of Helsinki, before any patients were enrolled.

### Immunoprecipitation (IP) and mass spectrometry (MS)

After cells were collected, they were lysed for 30 min on ice using lysis buffer supplemented with protease inhibitor cocktail (Beyotime, China). The cell lysates were incubated for 12- hours at 4 °C with rotation, and Protein A/G magnetic beads coated with an antibody against GCS1 (Santa Cruz, USA) or with IgG (Beyotime, China) as the negative control were added. After three washes with inhibitor lysate the next day, 100 µl of SDS-PAGE Sample Loading Buffer (1X) was added to each 20 µl volume of magnetic beads. The mixtures were subsequently heated for five minutes at 95 °C, and the beads were isolated by incubation for 10 s on a magnetic rack. The supernatant was then used for Western blotting.

### Ubiquitination assay

To extract total protein, cells were treated with 10 µM MG132 for 8 h after 48 h of transfection with the specified plasmid. To lyse the cells, Lysis Buffer with Protease Inhibitor Cocktail (Beyotime, China) was utilized. Myc-GRP78 was immunoprecipitated using protein A/G magnetic beads and an anti-Myc antibody. Then, an anti-HA antibody was used to detect ubiquitination of GRP78.

### Xenograft tumor model

Male, 4-week-old BALB/c nude mice were used in the investigation. There were six nude mice in each group. Each mouse was given an injection under the arm containing 100 µl per million cells to develop subcutaneous tumor models. The tumor volume was measured every seven days. After 28 days, the tumor was removed, weighed, photographed, and kept for additional study. This in vivo study was approved by the Animal Care and Use Committee of Nanjing Medical University (IACUC-2401023) and was conducted in accordance with ethical requirements.

### Statistical analysis

Each experiment was conducted independently three times. The experimental data were analyzed and presented as the means ± standard deviations (SDs) using GraphPad 9.0 (San Diego, USA) and SPSS v24.0 (Chicago, USA). ANOVA was used for comparisons among more than two groups, and Student’s t test was used for comparisons between two samples. The Kaplan-Meier (KM) method and the log-rank test were used for the survival analysis. To further elucidate the relationships between the GCS1 level and the clinicopathological features of CRC patients, the chi-square test was used. *P* < 0.05 was consider to indicate statistical significance. The sequences of the primers, and the information about the reagents and antibodies used are provided in the supplemental tables. Additional information is available in the supporting resources.

## Results

### GCS1 expression is elevated in CRC

**Fig. 1:**
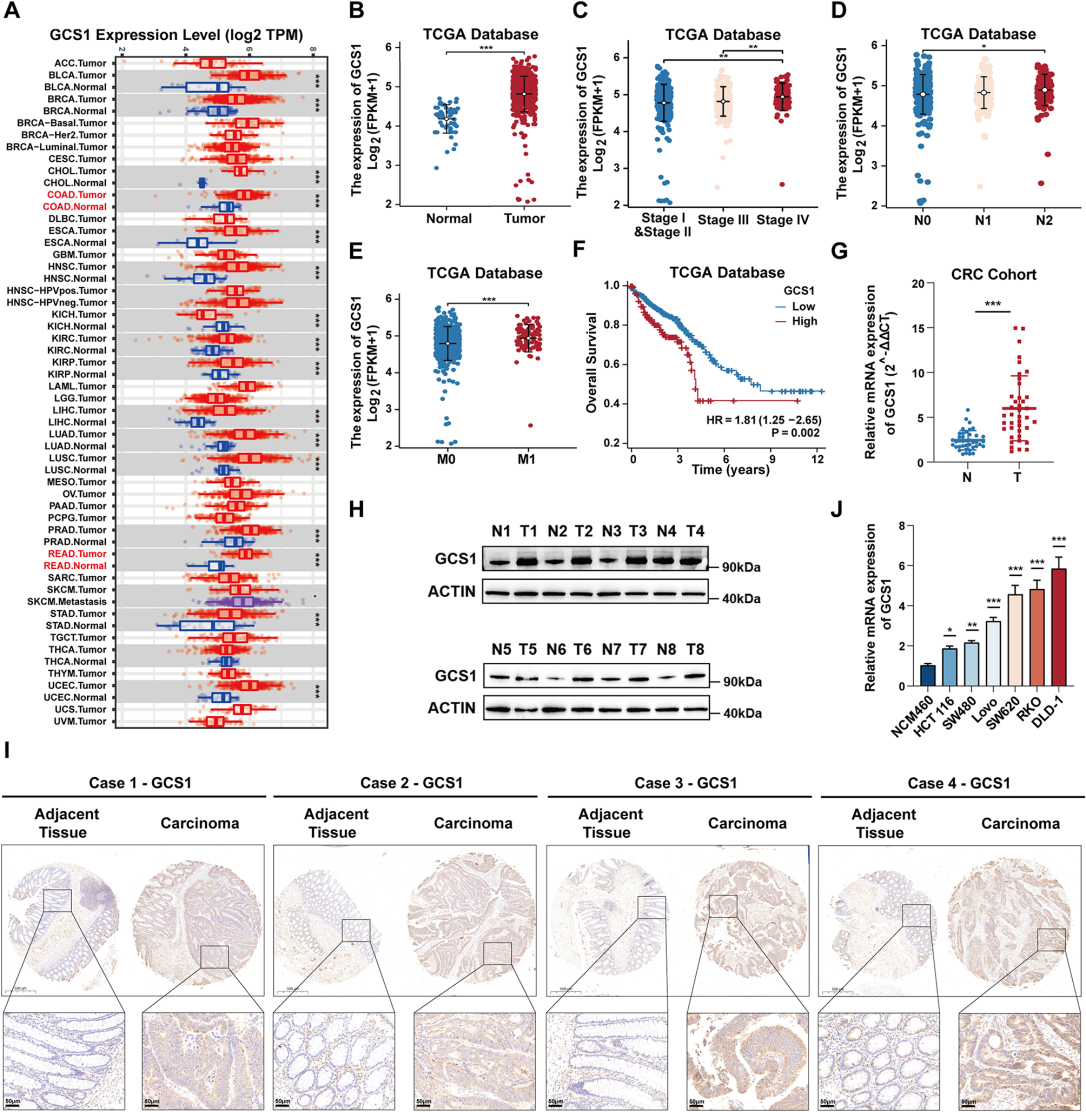
GCS1 is highly upregulated in CRC and indicates a poor prognosis. **A**: Expression of GCS1 across cancers according to the TIMER database. **B**: mRNA expression of GCS1 in CRC in the TCGA database. **C–E**: Associations of GCS1 expression with stage, lymph node metastasis status, distant metastasis status in the TCGA database. **F**: Association between GCS1 expression and OS in the TCGA database. **G**: GCS1 mRNA expression in clinical samples from our center (*n* = 40). **H**: GCS1 protein expression in clinical samples from our center (*n* = 8). **I**: Representative images of IHC staining for GCS1 in CRC tissues and paired adjacent normal tissues (*n* = 80). Scale bars, 100 μm. **J**: GCS1 expression in six different types of CRC cells and normal intestinal epithelial cells. The error bars indicate the mean ± SD of three independent experiments. * *P* < 0.05; ** *P* < 0.01; *** *P* < 0.001

In an early bioinformatic multi-omics analysis, our group was the first to show that GCS1 expression is up-regulated in CRC; however, the role and mechanism of GCS1 are still unknown. First, using the Tumor Immune Estimation Resource (TIMER) database, we found that the majority of tumor tissues had higher levels of GCS1 expression than did the counterpart normal tissues, particularly for cases of colon and rectal cancer (Fig. 1A). By examining the clinical data of CRC patients in The Cancer Genome Atlas (TCGA), we found that the expression level of GCS1 in CRC tissues was higher than that in normal intestinal epithelial tissues, and the same pattern was found in the UALCAN database (Fig. 1B, Figure S1A, B). Subsequent investigation revealed that GCS1 was up-regulated in patients with distant or lymphatic metastasis, as well as in patients with a high pathological stage (Fig. 1C-E). Next, we further explored the relationship between GCS1 and prognosis. The group with high GCS1 expression had a substantially shorter overall survival (OS) time than did the group with low GCS1 expression, as predicted (Fig. 1F). Additionally, the statistical results regarding disease-specific survival (DSS) and the progression- free interval (PFI) were consistent with the statistical results for OS (Figure S1C, D). Next, the Human Protein Atlas (HPA) database was analyzed, and the results provided additional evidence indicating that high expression of GCS1 in CRC tissues is linked to poor prognosis (Figure S1E).

Next, we used clinicopathological samples from our center to validate GCS1 expression at the transcriptional and translational levels. Consistent with our findings in public databases, GCS1 expression was noticeably increased in tumor tissues. First, the elevated RNA expression of GCS1 was confirmed using 40 pairs of CRC tissue samples (Fig. 1G). Then, high protein expression of GCS1 was detected in eight pairs of CRC tissue samples (Fig. 1H). A TMA was further used for verification, and the representative images showed that the expression of GCS1 in tumor tissues was greater than that in normal intestinal epithelial tissues (Fig. 1I). Analysis of the clinical data for 80 patients revealed that the expression level of GCS1 was strongly positively associated with TNM stage and lymph node metastasis status (Table S1). These preliminary results indicate that GCS1 tissues is substantially expressed in CRC and is strongly associated with prognosis and pathological stage in patients with CRC.

### GCS1 is essential for the malignant development of CRC in vitro

To further understand the biological role of GCS1 in CRC, the RNA and protein expression levels of GCS1 were measured in both the normal intestinal epithelial cell line NCM460 and various CRC tumor cell lines (Fig. 1J, Figure S1F). Then, DLD-1, RKO, and HCT 116 cells were chosen for further cell functional experiments. First, GCS1 short hairpin RNA (shRNA) was transfected into DLD-1 and RKO cells, and the overexpressed plasmid was transfected into HCT 116 cells. After transfection, GCS1 expression was significantly altered compared to that in the control group, as confirmed by measurement of RNA and protein expression (Figure S2A-F).

**Fig. 2:**
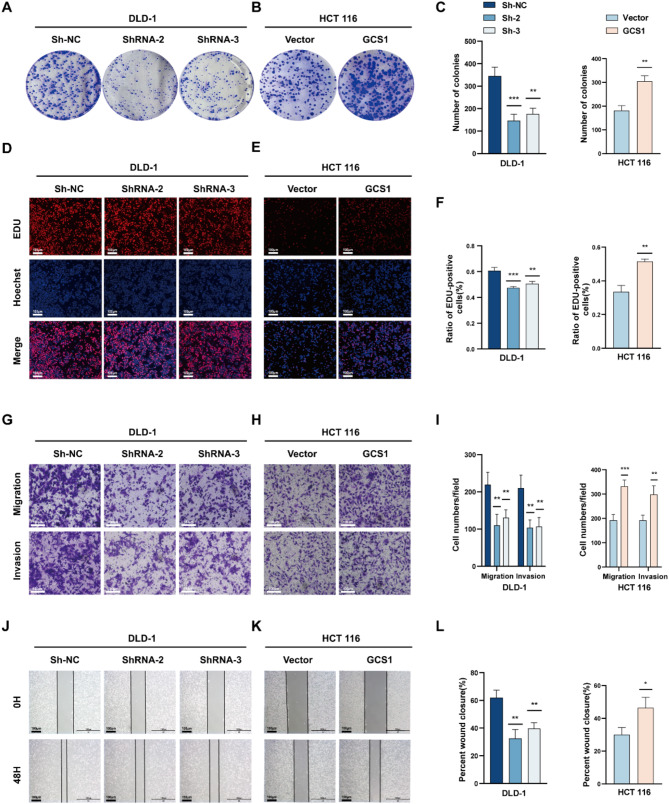
GCS1 accelerates the malignant progression of CRC in vitro. **A-C**: Representative images and the quantification of colonies of HCT 116 and DLD-1 cells transfected with the indicated plasmids or shRNAs. **D-F**: EdU-positive cell counts and representative images in both the abovementioned two cell lines. Scale bars, 100 μm. **G-I**: Representative images showing the quantities of migrated or invaded cells in the abovementioned two cell lines. Scale bars, 100 μm. **J-L**: Wound closure rate and representative images from the wound healing assay using both cell lines described above. Scale bars, 100 μm. The error bars indicate the mean ± SD of three independent experiments. * *P* < 0.05; ** *P* < 0.01; *** *P* < 0.001

Then, the cell proliferation ability of GCS1 was assessed using colony formation. The proliferation capacity of DLD-1 and RKO cells was significantly reduced when GCS1 was knocked down, whereas the proliferation capacity of HCT 116 cells was significantly increased when GCS1 was overexpressed (Fig. 2A-C, Figure S3D). Comparison of GCS1 knockdown DLD-1 and RKO cells to the corresponding control cells revealed that the number of EdU-positive cells was lower in the knockdown groups, but the number of EdU-positive HCT 116 cells was much greater in the GCS1 overexpression group than in the control group (Fig. 2D-F, Figure S3E). Moreover, the CCK-8 assay confirmed that GCS1 promoted cell proliferation (Figure S3A-C). The biological roles of GCS1 in invasion and migration were subsequently verified by Transwell and wound healing assays, which were conducted considering the notable differences in metastasis based on GCS1 expression, as explained above. The findings demonstrated that the migration and invasion capacities were greatly increased in the GCS1-overexpressing group, but were decreased in the GCS1 knockdown groups’ (Fig. 2G-L, Figure S3F-G). The results presented here imply that GCS1 accelerates the proliferation and metastasis of CRC cells in vitro.

### GCS1 alleviates ER stress and promotes malignant progression in vivo

**Fig. 3:**
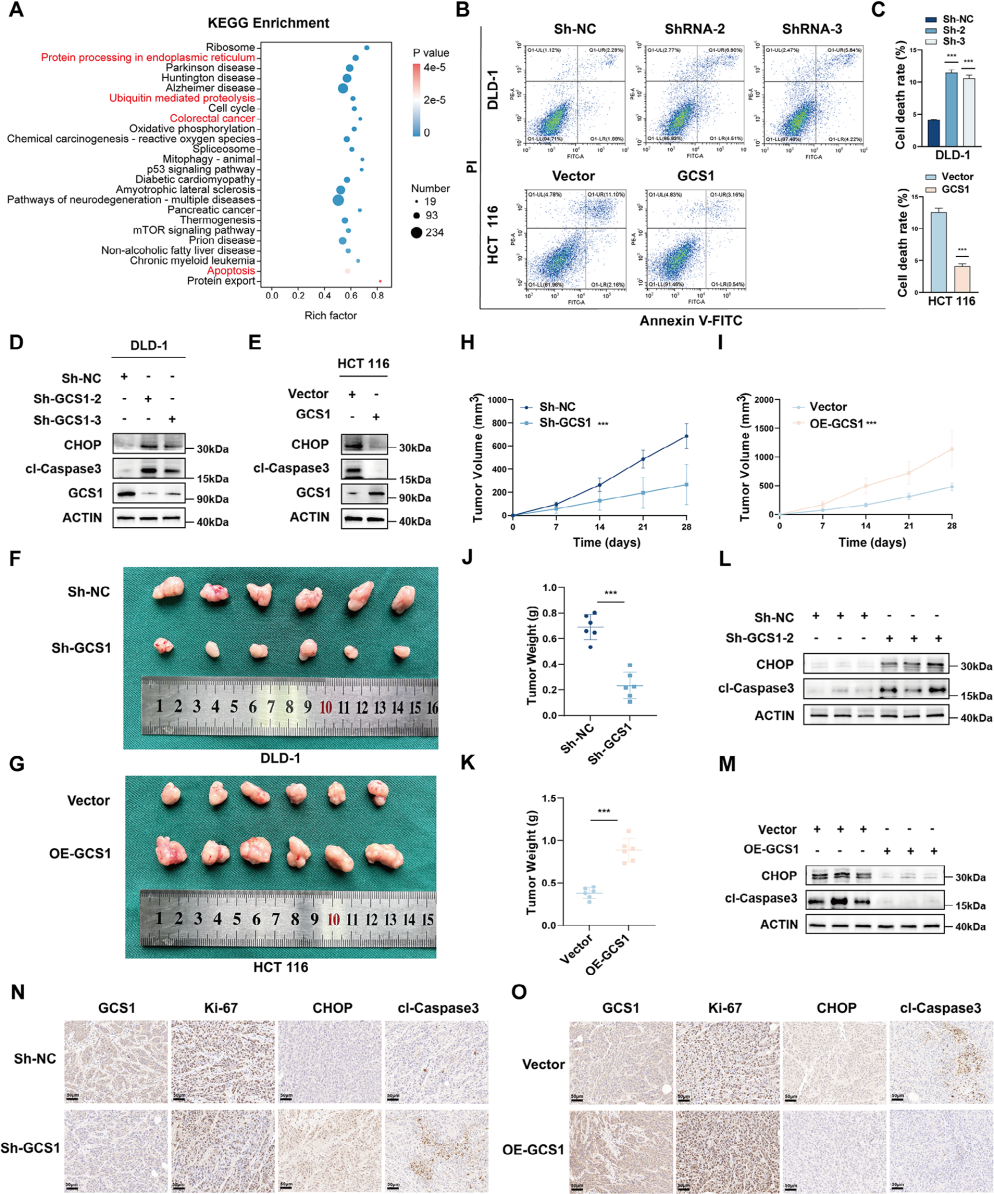
GCS1 alleviates ER stress-mediated apoptosis and promotes the malignant progression of CRC in vivo. **A**: KEGG enrichment analysis based on the RNA sequencing data (*n* = 3 replicates). **B**,** C**: Analysis of apoptosis in GCS1-knockdown DLD-1 cells and GCS1-overexpression HCT 116 cells using flow cytometry. **D**,** E**: The levels of cl-Caspase 3 and CHOP were measured in HCT 116 cells with GCS1 overexpression and DLD-1 cells with GCS1 knockdown. **F**: Representative images of tumors resected from nude mice injected with DLD-1 cells with stable knockdown of GCS1 expression (*n* = 6 mice). **G**: Representative images of tumors removed from nude mice injected with HCT 116 cells with stable overexpression of GCS1 (*n* = 6 mice). **H**,** I**: Calculated tumor volumes in the knockdown and overexpression groups. **J**,** K**: Calculated tumor weights in the knockdown and overexpression groups. **L**,** M**: The levels of CHOP and cl-Caspase 3 in tumors from the overexpression and knockdown groups were measured. **N**,** O**: Representative images of IHC staining in GCS1-knockdown and GCS1-overexpressed tumors. Scale bars, 50 μm. The error bars indicate the mean ± SD of three independent experiments. *** *P* < 0.001

Next, to further explore the mechanism of GCS1 in CRC, RNA sequencing was of HCT 116 cells in the GCS1 overexpression group and the control group (*n* = 3 replicates), and subsequent, Kyoto Encyclopedia of Genes and Genomes (KEGG) enrichment analysis revealed that GCS1 is involved mostly in protein processing in the ER, ubiquitin-mediated proteolysis, and the apoptotic pathway (Fig. 3A, Figure S4A). Previous studies have also shown that GCS1 can reduce ER stress. Thus, we hypothesized that GCS1 might control ER stress-mediated apoptosis in CRC.

Subsequently, flow cytometry (FCM) was used to demonstrate that compared with the corresponding control cells, HCT 116 cells overexpressing GCS1 had considerably reduced apoptosis rates, while DLD-1 and RKO cell with GCS1 knockdown had increased apoptosis rates (Fig. 3B, C; Figure S4B, C). Western blot (WB) analysis was subsequently employed to confirm the regulatory mechanisms linking GCS1 to ER stress and ER stress-mediated apoptosis at the translational level. WB analysis revealed that after GCS1 knockdown, the protein levels of the pro-apoptotic protein CHOP, produced during ER stress, and cleaved- Caspase 3 (cl-Caspase 3) were increased. However, in the GCS1 overexpression group, the levels of both CHOP and cl-Caspase 3 were greatly reduced (Fig. 3D, E). Then, we established tunicamycin (TM) treatment groups and control groups in both the knockdown and overexpression cell lines to further investigate the function of GCS1 in ER stress. The findings demonstrated that following TM treatment, GCS1 overexpression decreased the levels of CHOP and cl-Caspase 3, whereas GCS1 knockdown increased the levels of these proteins (Figure S4D, E). Subcutaneous xenograft models were used to investigate the oncogenic effect of GCS1 on CRC. DLD-1 cells transfected with shRNA (Sh-2) targeting GCS1, HCT 116 cells stably overexpressing GCS1, and the corresponding control cells were used to establish tumors. Four weeks after cell injection, the xenograft tumors were removed from the mice. Xenograft tumor growth was considerably inhibited by GCS1 deletion (Fig. 3F) but promoted by GCS1 overexpression (Fig. 3G). Additionally, the volume and weight of the tumors were measured (Fig. 3H-K). Collectively, the results indicated the tumor-promoting effects of GCS1 in vivo. Next, we extracted total protein from three randomly selected subcutaneous tumor-bearing nude mice from each group for WB analysis. The results were consistent with those observed in vitro (Fig. 3L, M). IHC staining revealed that GCS1 knockdown decreased the Ki-67 level but increased the CHOP and cl-Caspase 3 levels in xenograft tumors, while GCS1 overexpression increased the Ki-67 level but decreased the CHOP and cl-Caspase 3 levels (Fig. 3N, O). Taken together, these findings imply that GCS1 overexpression promotes the development of CRC by reducing ER stress-induced apoptosis and promotes the progression of CRC in vivo.

### GRP78 is the main target of GCS1

**Fig. 4:**
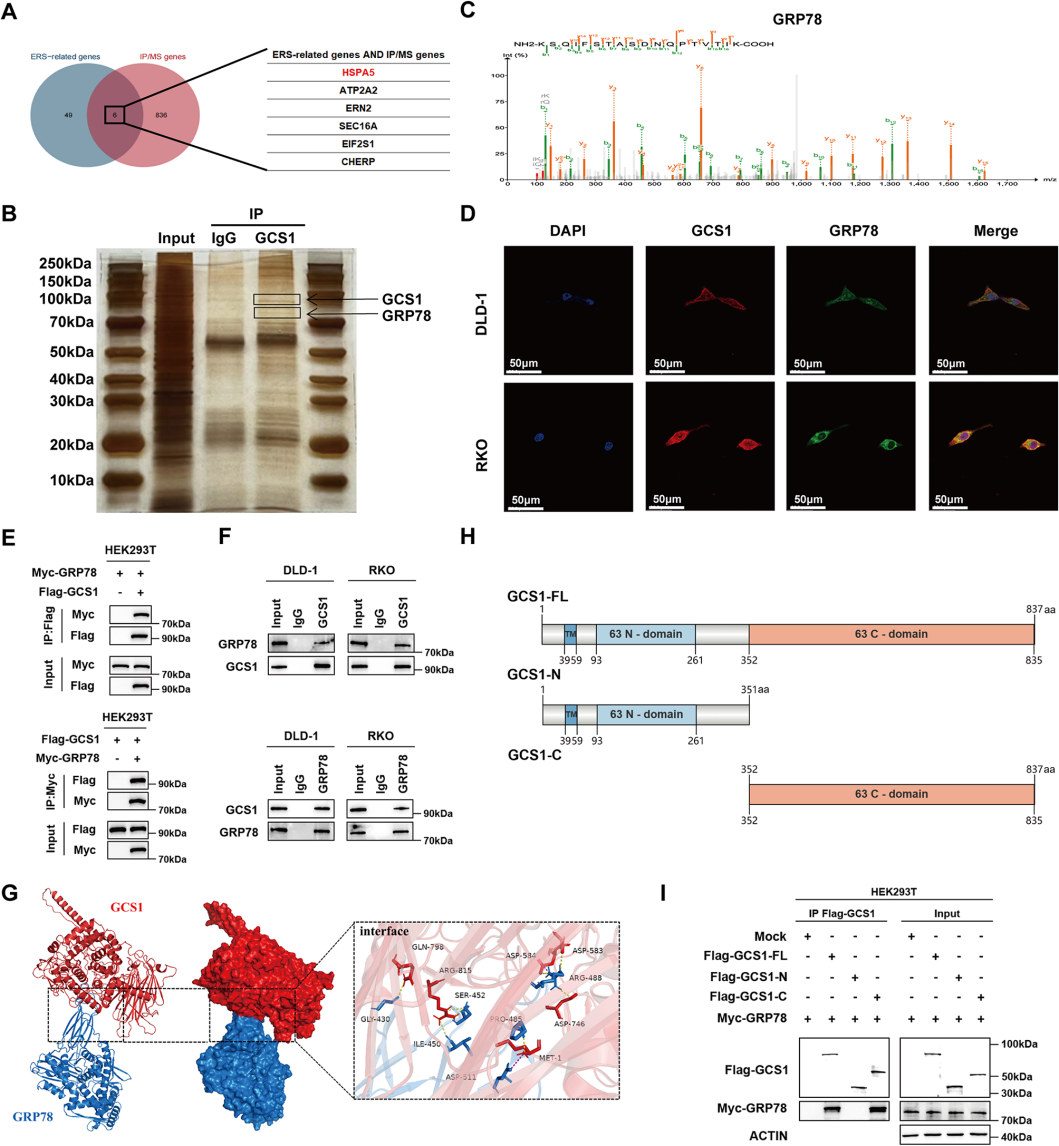
GCS1 interacts with GRP78. **A**: Six proteins identified from the overlap of proteins identified by IP-MS and ER stress-related proteins. **B**: Results of silver staining following IP of endogenous GCS1 in DLD-1 cells. **C**: Via IP and LC-MS analysis, specific peptides of GRP78 were identified. **D**: The subcellular location of GCS1 and GRP78 in DLD-1 and RKO cells was revealed by IF staining. Scale bars, 50 μm. **E**: The Co-IP results verified the binding between exogenous GCS1 and GRP78 in HEK293T cells. **F**: The binding of endogenous GCS1 and GRP78 was detected by Co-IP in DLD-1 and RKO cells. **G**: Protein-protein interaction analysis was performed to predict the binding sites of GCS1 and GRP78. **H**: Two plasmids expressing N- and C-terminal truncations were generated. A structural diagram of GCS1 is also presented. **I**: To identify the domains involved in the interaction between GCS1 and GRP78, HEK293T cells were separately transfected with plasmids expressing three flag-labeled proteins: GCS1-FL, GCS1-N, and GCS1-C

To further investigate how GCS1 regulates ER stress-mediated apoptosis, we isolated GCS1-interacting proteins from DLD-1 cells. Silver staining and MS were used to detect interacting proteins. Given that GCS1 can reduce ER stress, we determined the overlap between the proteins identified by IP-MS and the proteins associated with ER stress identified in the GeneCards database (Fig. 4A). Surprisingly, GRP78, also known as HSPA5 or Bip and a key protein regulating the translation of unfold proteins during ER stress, was ranked highest on the list of candidate proteins. Therefore, GRP78 was selected as a downstream protein of GCS1 for further study (Fig. 4B, C; Figure S5A). Next, the relationship between GCS1 and GRP78 was further verified. Immunofluorescence (IF) staining further confirmed that GCS1 and GRP78 were colocalized mainly in the cytoplasm of CRC cells (Fig. 4D). The exogenous binding of GRP78 to GCS1 in HEK293T cells was confirmed by coimmunoprecipitation (Co-IP) (Fig. 4E). In addition, DLD-1 and RKO cells were used to further validate the endogenous binding of GCS1 to GRP78 (Fig. 4F). Subsequently, we used the protein-protein docking approach to predict the binding domain between GCS1 and GRP78 to gain a deeper understanding of their interaction, and the results are displayed in Fig. 4G. Then, we constructed a GCS1 truncation plasmid, (in the related experiments, Flag-GCS1-FL refers to the unmodified GCS1 protein). The sequences encompassing amino acids (aa) 1-351 and 352–837 were truncated and the resulting proteins were named Flag-GCS1-N and Flag-GCS1-C, respectively (Fig. 4H). The Flag-GCS1-FL/N/C and Myc-GRP78 plasmids were transfected into HEK293T cells. Co-IP revealed that GRP78 bound primarily to the C terminus of GCS1 (Fig. 4I).

### GCS1 stabilizes the GRP78 protein by inhibiting its ubiquitination

**Fig. 5:**
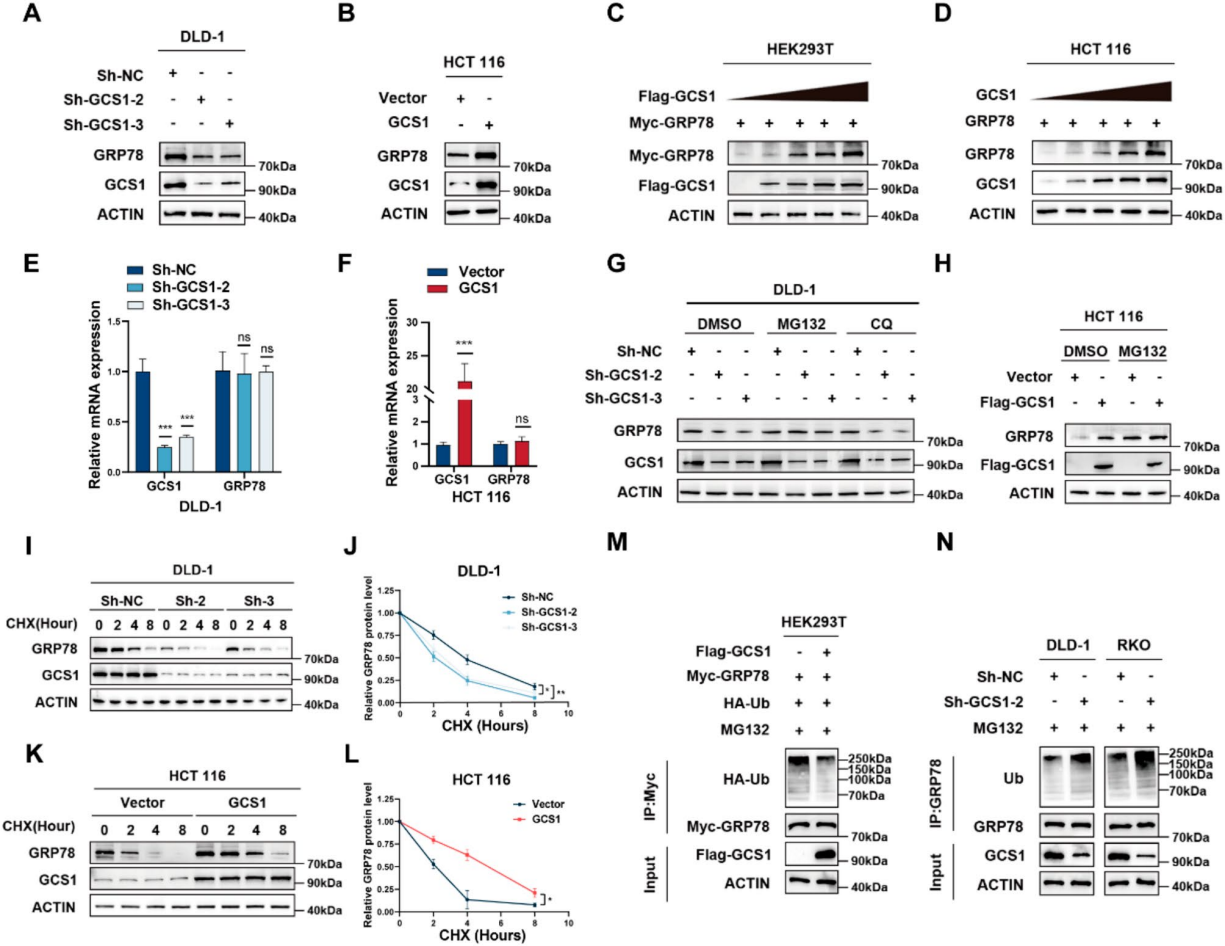
GCS1 inhibits GRP78 degradation through the ubiquitination pathway. **A**,** B**: GCS1 was knocked down in DLD-1 cells or overexpressed in HCT 116 cells, and total protein was extracted to measure the expression of GCS1 and GRP78. **C**: The expression levels of GCS1 and GRP78 were measured after transfecting HEK293T cells with the Myc-GRP78 plasmid and the Flag-GCS1 plasmid. **D**: To measure the expression levels of GCS1 and GRP78, HCT 116 cells were transfected with various amounts of the GCS1 overexpressed plasmid. **E**,** F**: Changes in GRP78 expression following knockdown or overexpression of GCS1 at the transcriptional level. **G**,** H**: GRP78 expression was measured in DLD-1 cells with GCS1 knockdown and HCT 116 cells with GCS1 overexpression, following treatment with MG132 (10 µM) or CQ (50 µM). **I-L**: Effect of CHX (100 µg/mL) on GRP78 expression after GCS1 overexpression in HCT 116 cells and after GCS1 knockdown in DLD-1 cells. **M**: HEK293T cells transfected with Flag-GCS1, Myc-GRP78, HA-Ub, or empty plasmid following treatment with 10 µM MG132 were used to study GRP78 ubiquitination. **N**: After treatment with 10 µM MG132, GRP78 ubiquitination was assessed in DLD-1 and RKO cells stably transfected with Sh-2 or Sh-NC. The error bars indicate the mean ± SD of three independent experiments. ns, not significant; * *P* < 0.05; ** *P* < 0.01; *** *P* < 0.001

The next task was to determine whether GCS1 can control GRP78 expression. We discovered that GRP78 protein expression was also decreased in GCS1 knockdown cells (Fig. 5A, Figure S5B) but was significantly increased in GCS1-overexpression cells (Fig. 5B). Moreover, GCS1 overexpression led to a dose-dependent increase in GRP78 protein expression (Fig. 5C, D). However, neither overexpression nor knockdown of GCS1 had no effect on GRP78 mRNA expression. (Fig. 5E, F; Figure S5D).

Given the RNA sequencing findings previously discussed, which also confirmed that GCS1 is involved in the ubiquitin-proteasome degradation process, and a previous report indicating that USP11 can block GRP78 degradation and promote tumor progression [29], we postulate that GCS1 may post-transcriptionally control the GRP78 protein level. To further determine whether GCS1 inhibits GRP78 degradation through the lysosomal or the proteasomal pathway, DLD-1 cells were used for experiments. Protein was extracted from cells treated with the lysosome inhibitor chloroquine (CQ) or the proteasome inhibitor MG132 for WB analysis. Treatment with the lysosome inhibitor CQ did not prevent GRP78 degradation. Moreover, there was no significant change in the expression of GRP78 in the MG132 treatment group (Fig. 5G). We verified these results in RKO and HCT 116 cells, and the results were parallel to or opposite to those in DLD-1 cells, respectively (Fig. 5H, Figure S5C). These results indicated that GCS1 inhibited the degradation of GRP78 via the ubiquitin-proteasome pathway. Next, we used the protein synthesis inhibitor cycloheximide (CHX) to study the effect of GCS1 on the degradation of the GRP78 protein. GCS1 overexpression significantly inhibited the degradation of endogenous GRP78, and after GCS1 knockdown, the degradation of endogenous GRP78 was significantly increased. We also verified the effect of GCS1 overexpression on the degradation of exogenous GRP78 in HEK293T cells, and the results were consistent with those for endogenous GRP78, indicating that GCS1 can prolong the half-life of GRP78 (Fig. 5I-L, Figure S5E-H). The degradation of GRP78 was then inhibited by transfecting HA-labeled ubiquitin into cells treated with MG132. The GCS1 overexpression group showed a decrease in the abundance of ubiquitinated GRP78 in the complex immunoprecipitated with an anti-GRP78 antibody (Fig. 5M). On the other hand, abundance of precipitated ubiquitinated GRP78 was increased in the GCS1 knockdown group (Fig. 5N). In conclusion, these findings imply that GCS1 binds to GRP78 and, via the ubiquitin proteasome pathway, decreases the ubiquitination level of GRP78, preventing its degradation.

### GCS1 recruits USP10 to mitigate GRP78 ubiquitination

**Fig. 6:**
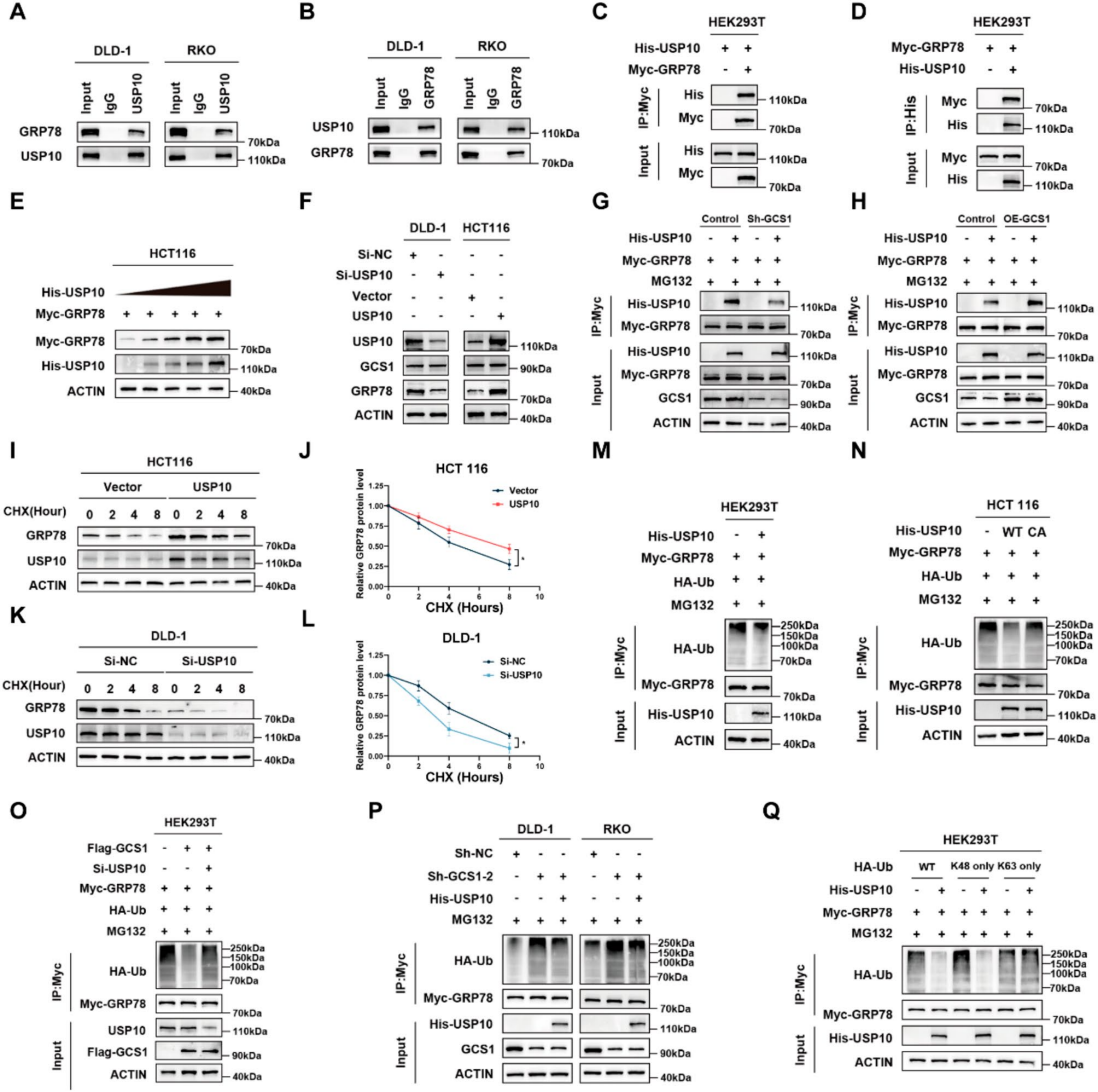
GCS1 recruits USP10 to reduce the K48-linked ubiquitination of GRP78. **A**,** B**: The binding of endogenous USP10 and GRP78 in DLD-1 and RKO cells was validated. **C**,** D**: Validation of exogenous USP10 and GRP78 binding in HEK293T cells. **E**: The GRP78 expression level increased with increasing concentrations of the USP10 overexpression plasmid. **F**: USP10 was downregulated in DLD-1 cells and overexpressed in HCT 116 cells, and GCS1 and GRP78 expression were measured. **G**,** H**: GCS1 was downregulated in DLD-1 cells and overexpressed in HCT 116 cells, and the ability of USP10 and GRP78 to bind to each other was determined. **I-L**: Effect of CHX (100 µg/mL) on GRP78 protein expression after USP10 overexpression in HCT-116 cells and USP10 knockdown in DLD-1 cells. **M**: HEK293T cells were transfected with His-USP10 to measure the level of ubiquitinated Myc-GRP78. **N**: Following the transfection of His-USP10 and His-USP10 (C424A) into HEK293T cells, the level of ubiquitinated GRP78 was measured. **O**: After HEK293T cells was transfected with Flag-GCS1, Myc-GRP78, and Si-USP10, the level of ubiquitinated GRP78 was measured. **P**: After USP10 was overexpressed in DLD-1 and RKO cells in which GCS1 was stably knocked down, the level of ubiquitinated GRP78 was measured. **Q**: Following the transfection of His-USP10, HEK293T cells were transfected with the K48-only and K63-only ubiquitin variants, and the level of ubiquitinated GRP78 was compared. The error bars indicate the mean ± SD of three independent experiments. * *P* < 0.05

The above findings demonstrated that while GCS1 is not a ubiquitination enzyme, it does bind to GRP78 and control its level of ubiquitination. Thus, we hypothesized that GCS1 can decrease GRP78 degradation by recruiting DUBs. USP10, the DUB with the greatest binding strength identified by MS, was chosen as a candidate molecule for additional confirmation (Figure S6A). The binding among endogenous and exogenous GCS1, GRP78, and USP10 was then confirmed by Co-IP. GRP78 was present in the USP10 immunoprecipitate, and the USP10 protein was also present in the GRP78 immunoprecipitate (Fig. 6A-D). We also examined the binding of both endogenous and exogenous USP10 and GCS1 (Figure S6B-E). Neither knockdown nor overexpression of USP10 significantly changed the transcript level of GRP78 (Figure S6F-H); gradual overexpression of USP10 led to a gradual increase in the protein level of GRP78 (Fig. 6E, Figure S6I) but did not affect the expression level of GCS1 (Fig. 6F). Further study revealed that the binding ability of USP10 and GRP78 was reduced when GCS1 was downregulated but increased when GCS1 was overexpressed (Fig. 6G, H). Next, we used the protein synthesis inhibitor CHX to study the effect of USP10 on the degradation of the GRP78 protein. Overexpression of USP10 significantly inhibited the degradation of both endogenous and exogenous GRP78 (Fig. 6I, J; Figure S6J, K). Conversely, after USP10 knockdown, the degradation of endogenous GRP78 was significantly increased (Fig. 6K, L, L and M). These results indicated that overexpression of USP10 significantly inhibited the degradation of GRP78. Further experimental results showed that overexpression of USP10 significantly reduced the ubiquitination level of GRP78 (Fig. 6M). Subsequently, to investigate whether the deubiquitinase activity of USP10 is involved in the regulation of GRP78 degradation, USP10 (C424A), a deubiquitinase-inactive mutant of USP10, was transfected into HEK293T and HCT 116 cells, and the results showed that USP10 (C424A) was unable to stabilize and deubiquitinate GRP78 (Fig. 6N, Figure S6N, O). These results indicated that the deubiquitinase activity of USP10 was important for the stability of GRP78. Furthermore, the inhibitory effect of exogenous GCS1 on GRP78 ubiquitination was reduced by USP10 knockdown (Fig. 6O). Consequently, USP10 overexpression in DLD-1 and RKO cells reversed the GCS1 knockdown-induced increase in GRP78 ubiquitination (Fig. 6P). We transfected HA-tagged Ub variants, including wild-type (WT), K48-only (ubiquitin with only the Lys48 residue intact), and K63-only (ubiquitin with only the Lys63 residue intact) Ub, to further determine the specific ubiquitination linkage responsible for the degradation of GRP78. We found that USP10 regulated the stability of GRP78 through K48-linked polyubiquitination (Fig. 6Q). In conclusion, GCS1 can decrease the ubiquitination level of GRP78 by increasing the ability of USP10 to cleave the K48-linked polyubiquitin chains of the protein.

### GCS1 alleviates ER stress and promotes CRC progression by regulating GRP78

**Fig. 7:**
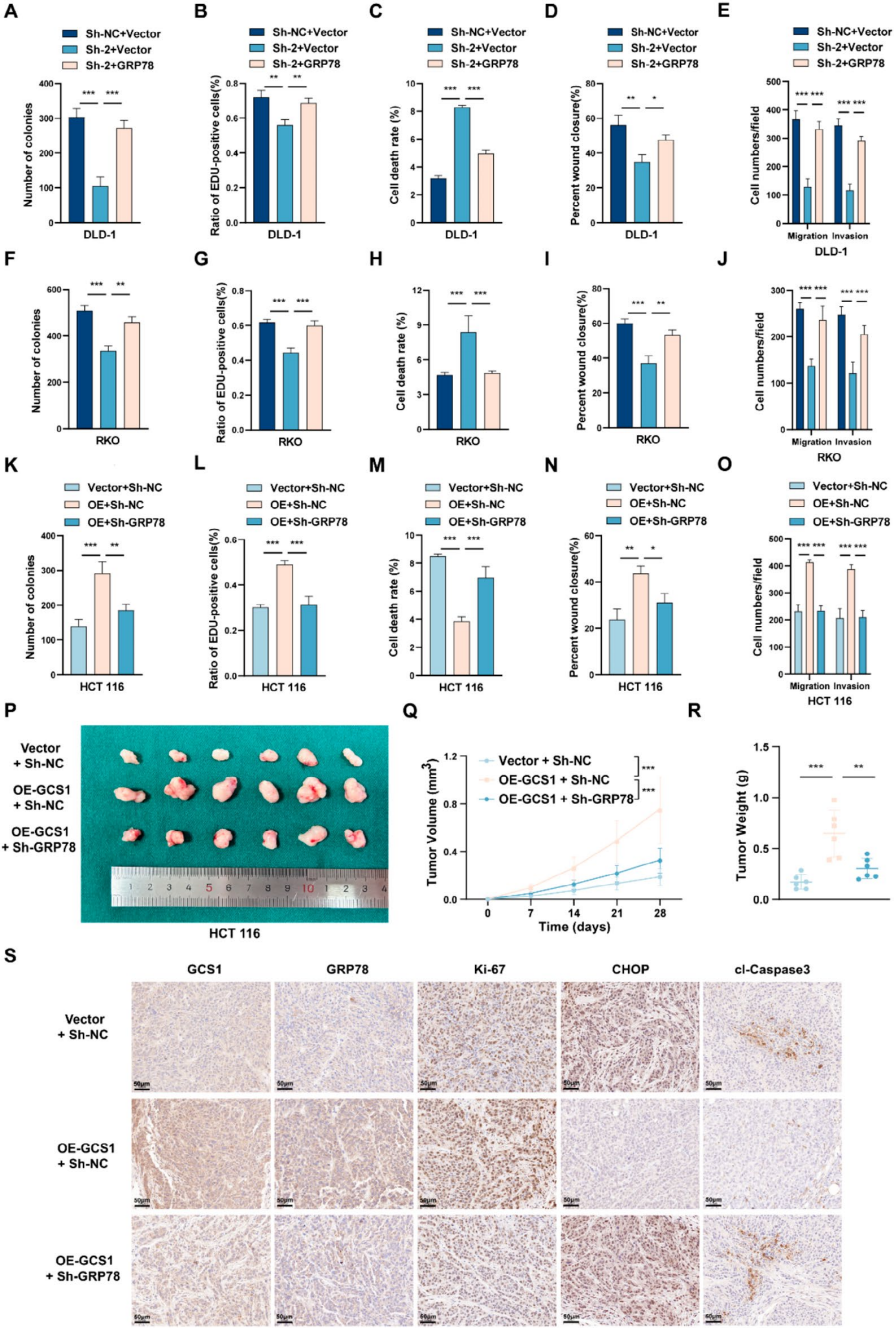
GRP78 is the functional downstream protein of GCS1. **A–E**: Effects of GRP78 overexpression on colony formation, EdU incorporation, apoptosis, wound healing, and Transwell migration in DLD-1 cells with stable knockdown of GCS1. **F–J**: Impacts of GRP78 overexpression on the abovementioned parameters in RKO cells with stable knockdown of GCS1. **K-O**: The impact of GRP78 knockdown on the abovementioned parameters was investigated in HCT 116 cells with stable GCS1 overexpression. **P**: Typical images of subcutaneous tumors harvested from nude mice in the three groups (*n* = 6 mice). **Q**,** R**: Tumor volumes and weights in the three groups. **S**: Three sets of representative IHC images showing the expression of related proteins. The error bars indicate the mean ± SD of three independent experiments. * *P* < 0.05; ** *P* < 0.01; *** *P* < 0.001

Further studies were conducted in vitro and in vivo to confirm the role of GRP78 in GCS1-mediated promotion of CRC progression. GRP78 was knocked down in HCT 116 cells with stable GCS1 overexpression and overexpressed in DLD-1 and RKO cells with stable GCS1 knockdown. The results of cell proliferation assays, such as colony formation, EdU incorporation, and CCK8 assays, confirmed that GRP78 overexpression mitigated the GCS1 knockdown-induced reduction in the proliferation ability of CRC cells. However, GRP78 silencing had the opposite effect on cells overexpressing GCS1. Moreover, flow cytometry was used to determine whether GRP78 is required for the ability of GCS1 to control apoptosis. The results confirmed that in cells with GCS1 overexpression, GRP78 knockdown partially restored the capacity of these cells to undergo apoptosis. GCS1 knockdown, on the other hand, had the opposite effect. The migration and invasion capacities were assessed using Transwell and wound healing assays. Similar to the findings in the cell proliferation assays, overexpression of GRP78 can partially reverse the decreases in cell migration and invasion observed after GCS1 knockdown (Fig. 7A-O, Figure S7A-F and Figure S8A-B).

The indispensable involvement of GRP78 in the GCS1-mediated promotion of CRC progression was further confirmed by representative images of subcutaneous xenograft tumors in nude mice, statistical analysis of the tumor weight and volume, and representative immunohistochemical images of tumors (Fig. 7P-S). Based on the aforementioned findings, GCS1 depends on GRP78 to suppress apoptosis mediated by ER stress and hence accelerate the development of CRC.

### The expression levels of GCS1 with poor prognosis and GRP78 are increased in CRC and are positively correlated

**Fig. 8:**
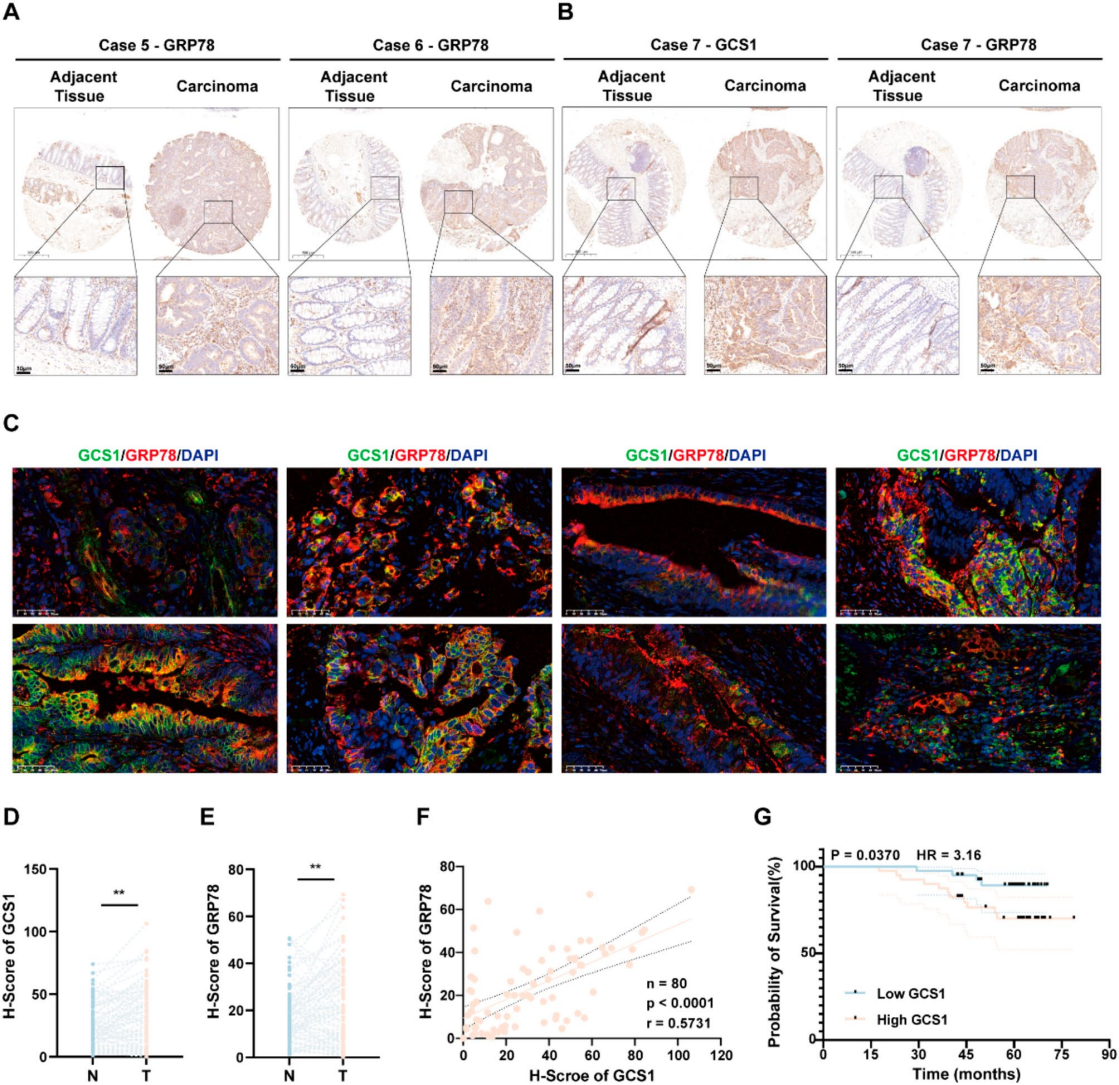
Increased GCS1 expression is associated with increased GRP78 expression and can be used to predict poor outcomes in CRC patients. **A**: Representative images showing GRP78 expression in paired CRC and adjacent normal tissue samples. **B**: Representative images showing GCS1 and GRP78 expression in paired CRC and adjacent normal tissue samples. **C**: Representative images showed the results of co-localization of GCS1 and GRP78 in paired CRC and adjacent normal tissue samples. **D-F**: The H-scores of GCS1 expression were positively correlated with the H-scores for GRP78 expression in individual CRC patients. **G**: Kaplan-Meier curves of the OS time of CRC patients stratified by GCS1 protein expression. ** *P* < 0.01

We verified that GCS1 overexpression results in GRP78 deubiquitination and stabilization, which influences ER stress-mediated apoptosis and promotes CRC growth and metastasis. Using TMA data from our center, we further confirmed the relationship between the expression of GCS1 and that of GRP78 to validate our hypothesis. As predicted, considerable upregulation of GRP78 expression was detected in tumor tissues compared with the matched normal intestinal epithelial tissues in CRC patients (Fig. 8A, B; Figure S9A, B). Meanwhile, IF co-localization also demonstrated cytoplasmic co-localization of GCS1 and GRP78 in CRC tissues (Fig. 8C, Figure S9C, D). In addition, GCS1 and GRP78 expression exhibited a significant correlation (*r* = 0.5731) (Fig. 8D-F). Last, and perhaps most importantly, the overall survival time was shorter in patients with high expression of GCS1 than in those with low expression (Fig. 8G). Overall, analysis of our CRC clinical data strongly confirmed our experimental findings.

## Discussion

Previous research has demonstrated that the ER stress-induced UPR is essential for the initiation and control of CRC. However, the exact regulatory systems and interaction networks involved in vivo are currently unknown [12, 30]. We previously found that GCS1 expression is highly increased in CRC [13]. The purpose of the present work was to clarify the processes by which GCS1 controls the progression of CRC. In this work, we showed that GCS1 is substantially expressed in CRC and that a higher level of GCS1 expression is associated with a shorter survival time. We found that GCS1 inhibits ER stress-mediated apoptosis while promoting the growth and metastasis of CRC. Zhou et al. revealed that GCS1 promotes CRC metastasis through the Notch signaling pathway, consistent with our findings [31]. The relationship between GCS1 and GRP78 was then revealed, and it was demonstrated that GCS1 recruits USP10 to increase GRP78 stability by decreasing its K48-linked ubiquitination, which in turn mitigates ER stress-mediated apoptosis and promotes CRC growth and metastasis. In conclusion, we present a unique mechanism by which GCS1 mediates ER stress in CRC and propose that GCS1 may be a therapeutic target.

GCS1, a member of the glycosyl hydrolase 63 family, primarily supports the correct folding of proteins, and has previously been partially linked to viral infections and rare genetic abnormalities [32–35]. According to recent research, GCS1 expression is increased in the brain microvessels of patients with Alzheimer’s disease and controls the migration, proliferation, and differentiation of Schwann cells [15, 16]. Furthermore, GCS1 can mitigate ER stress caused by free fatty acids in mammalian cells [17]. However, the mechanisms by which GCS1 regulates cancers, especially CRC, remain unknown. We are the first to show that GCS1 inhibits apoptosis and promotes CRC cell proliferation and metastasis by modulating ER stress. Our research focused on the relationship between GCS1 and ER stress. Through colony formation, EdU incorporation, and Transwell assays, we showed that GCS1 facilitates the migration, invasion, and proliferation of CRC. Flow cytometric analysis revealed that GCS1 suppresses CRC cell apoptosis. Further evidence supporting the capacity of GCS1 to promote cell proliferation and prevent ER stress-mediated apoptosis was obtained in mouse xenograft models in vivo.

Through a variety of mechanisms, including invasion, proliferation, drug resistance, and tumor microenvironment regulation, ER stress can impact the course of CRC and the response to treatment [18, 36–40]. Unfolded proteins are bound by GRP78, a crucial regulator of cellular homeostasis, which promotes their folding and decreases ER stress. Inhibiting apoptosis and promoting tumor cell proliferation and metastasis are important functions of GRP78 [41, 42]. For example, PPI suppresses GRP78 expression and subsequent CHOP ubiquitination to prevent non-small cell lung cancer cells from undergoing apoptosis [43]. By promoting the ubiquitination and degradation of GRP78, SCNN1B prevents the growth and metastatic spread of stomach cancer [44]. In colorectal cancer, GRP78 expression increases gradually from normal tissue to adenoma tissue to carcinoma tissue [45]. In light of our earlier discovery that GCS1 controls ER stress in CRC, we used IP-MS to identify ER stress-related proteins. GRP78 was the most highly enriched protein, indicating that GCS1 may interact with GRP78 to control ER stress in CRC. According to KEGG enrichment analysis, ubiquitin mediated proteolysis was primary pathway driving GCS1 enrichment. We thus hypothesized that GCS1 overexpression decreases the level of ubiquitinated GRP78, which in turn stimulates the binding of GRP78 to unfolded proteins. This process facilitates the degradation of these misfolded proteins and reduces ER stress.

The increased GCS1 expression after MG132 treatment decreased GRP78 ubiquitination, according to subsequent investigations. Since GCS1 is not a DUB, we postulated the existence of a regulatory molecule that facilitates the deubiquitination of GRP78. Additional MS analysis revealed nine DUBs, which USP10 was the most enriched. USP10, a member of the USP subfamily, has been extensively studied in tumors. Prior research has demonstrated that USP10 promotes the growth of tumors by interacting with a variety of substrate proteins, including N1ICD, DDX21, MOF and HDAC7 [46–49]. According to another study, USP10 functions as a scaffold protein in esophageal squamous cell carcinoma, increasing DNA repair activity and decreasing the lethality of ionizing radiation [50]. In our study, USP10 functions as a scaffold protein in the GCS1–GRP78 pathway, promoting the cleavage of K48-linked polyubiquitin chains of GRP78. DUBs control the activity, stability, and location of target proteins, which in turn control the progression of CRC [51–53]. In this work, we first showed that USP10 binds to GRP78 and GCS1, which increases the ability of USP10 to cleave the K48-linked polyubiquitin chains of GRP78 and, in turn, increases GRP78 stability under the condition of increased GCS1 expression. However, the precise binding sites are for GCS1, GRP78, and USP10 are still unknown.

We next conducted both in vitro and in vivo rescue experiments, which demonstrated that GRP78 inhibition partially counteracted the effects of GCS1 on CRC cell proliferation and metastasis and on ER stress-induced apoptosis. These findings imply that GCS1 controls GRP78 expression, which in turn affects CRC malignancy. Furthermore, the result of TMA immunohistochemistry showed that in contrast to normal intestinal epithelial tissues, colorectal tumor tissues expressed high levels of both GSC1 and GRP78. Moreover, we found that the expression levels of GRP78 and GCS1 were significantly correlated. Meantime, we performed tissue IF analysis, and the results indicated that GCS1 and GRP78 were colocalized in the tissues. These data support our in vivo and in vitro results. Comparison of clinical data between the GCS1 high expression group and the low expression group, significant differences were observed for only TNM stage and lymphatic metastasis status. However, the difference in distant metastasis status found in the public database was not found in our TMA samples, possibly because of the small sample size of patients with metastasis in our study. We intend to increase the size of the clinical sample in the future in order to investigate the relationship between GCS1 expression and metastasis in more detail.

**Fig. 9:**
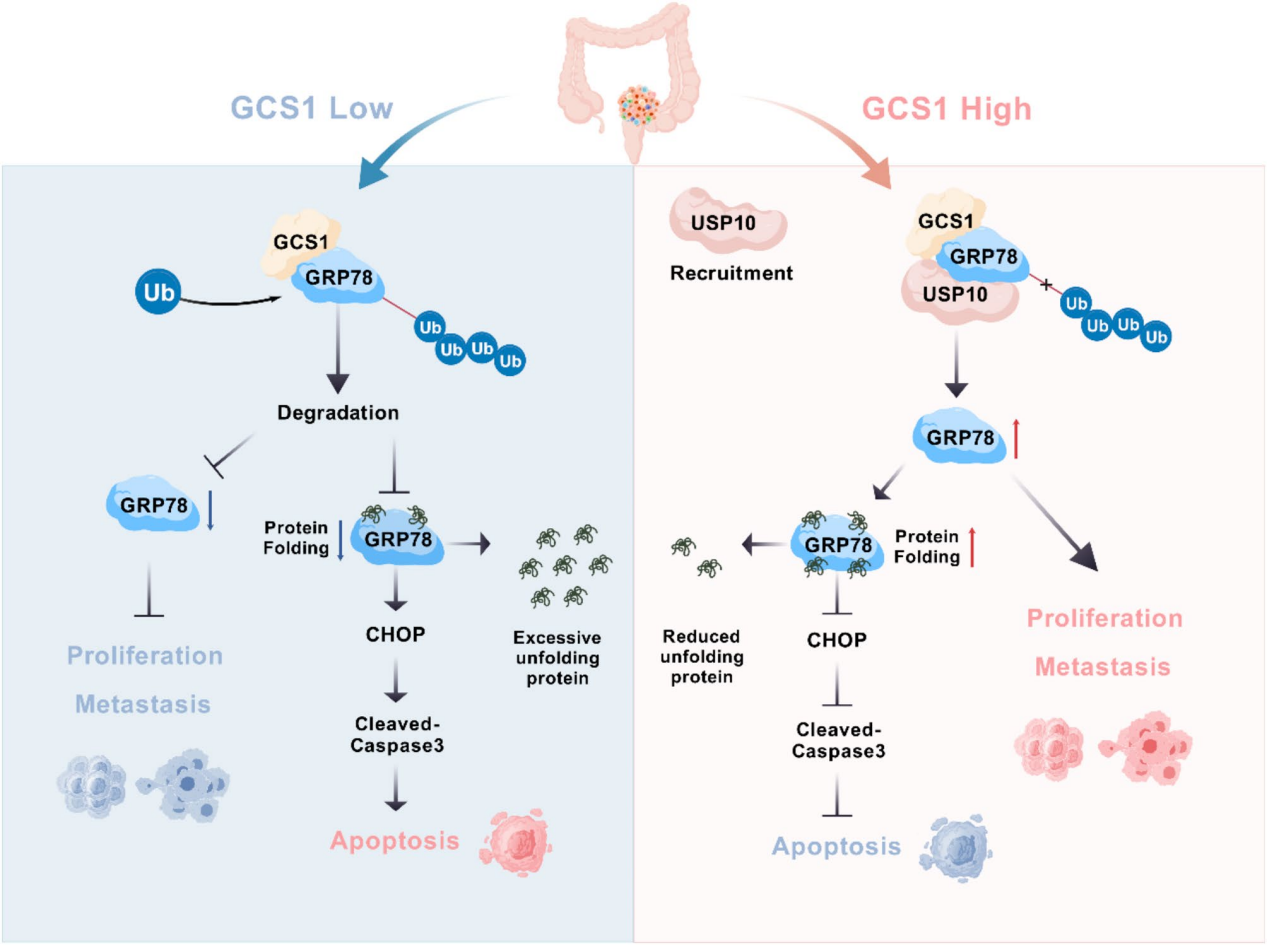
A schematic diagram of this study. Increased GCS1 decreases ER stress-mediated apoptosis and promotes proliferation and metastasis in CRC

Based on our current research, high expression of GCS1 is associated with poor prognosis in CRC patients. Furthermore, GCS1 promotes CRC cell proliferation and metastasis while mitigating ER stress-mediated apoptosis. Mechanistically, GCS1 recruits USP10 to cleave the K48-linked polyubiquitin chains of GRP78, increasing GRP78 stability, decreasing the production of the apoptotic protein CHOP during ER stress, and eventually promoting the malignant progression of CRC (Fig. 9). Our findings provide support the idea that targeting GCS1 could be a successful treatment approach for CRC.

### Web links and URLs

1. TIMER database: https://cistrome.shinyapps.io/timer/.
2. UALCAN database: https://ualcan.path.uab.edu/index.html.
3. HPA database: https://www.proteinatlas.org/.
4. GeneCards database: https://www.genecards.org/.

## Electronic supplementary material

Below is the link to the electronic supplementary material.

Supplementary Material 1

## Data Availability

The data in the current study are available from the corresponding author on reasonable request.

## Abbreviations

CRC: Colorectal Cancer
ER: Endoplasmic Reticulum
GCS1: Glucosidase I
UPR: Unfolded Protein Response
DUB: Deubiquitinating Enzyme
TMA: Tissue Microarray
IP: Immunoprecipitation
MS: Mass Spectrometry
SD: Standard Deviation
KM: Kaplan‒Meier
TIMER: Tumor IMmune Estimation Resource
TCGA: The Cancer Genome Atlas
OS: Overall Survival
DSS: Disease-Specific Survival
PFI: Progression-Free Interval
HPA: Human Protein Atlas
shRNA: Short Hairpin RNA
KEGG: Kyoto Encyclopedia of Genes and Genomes
WB: Western Blot
IHC: Immunohistochemistry
IF: Immunofluorescence
cl-Caspase 3: Cleaved-Caspase 3
TM: Tunicamycin
ERS: Endoplasmic Reticulum Stress
Co-IP: Coimmunoprecipitation
CQ: Chloroquine
CHX: Cycloheximide
