# Supplementary material for: Recruitment of USP10 by GCS1 to deubiquitinate GRP78 promotes the progression of colorectal cancer via alleviating endoplasmic reticulum stress
Source: J Exp Clin Cancer Res. 2024 Sep 13;43:261. doi: 10.1186/s13046-024-03176-8 (PMC11396530; doi:10.1186/s13046-024-03176-8)
Supplement: Supplementary file 1 — Supplementary Material 1 [file 13046_2024_3176_MOESM1_ESM.docx]

**Recruitment of USP10 by GCS1 to deubiquitinate GRP78 promotes the progression of colorectal cancer via alleviating endoplasmic reticulum stress**

Yang Chen^1,2†^, Hengyang Shen^1,2†^, Zhenling Wang^1,2†^, Changzhi Huang^1,2^, Hongqiang Zhang^1,2^, Yu Shao^1,2^, Ying Tong^1,2^, Lei Xu^1,2^, Yunfei Lu^1,2^, Zan Fu^1,2*^

1.Department of General Surgery, The First Affiliated Hospital of Nanjing Medical University, Nanjing, Jiangsu, China.

2.The First School of Clinical Medicine, Nanjing Medical University, Nanjing, Jiangsu, China.

***Correspondence author**

Zan Fu, Ph.D. Department of General Surgery, The First Affiliated Hospital of Nanjing Medical University, 300 Guangzhou Road, Nanjing, Jiangsu, 210009, P.R. China;

E-mail: fuzan1971@njmu.edu.cn;

^†^These authors contribute equally to this work.

**Table of contents:**

Supplementary materials and methods

Supplementary figure 1

Supplementary figure 2

Supplementary figure 3

Supplementary figure 4

Supplementary figure 5

Supplementary figure 6

Supplementary figure 7

Supplementary figure 8

Supplementary figure 9

Supplementary table 1

Supplementary table 2

Supplementary table 3

Supplementary table 4

**Supplementary materials and methods**

**Cell lines and cultures**

The human CRC cell lines used included HCT 116, SW620, DLD-1, RKO, Lovo, and SW480; the normal colon epithelial cell line used was NCM460. Additionally, the HEK293T cell line was used. These cells were acquired from the Chinese Academy of Sciences' Shanghai Institute of Biochemistry and Cell Biology, which is located in Shanghai, China. Every cell line was cultivated in the appropriate medium in an incubator with 5% CO2 and a temperature of 37°C.

**Cell transfection**

Both the relevant negative control Sh-NC and the short hairpin RNAs (Sh-RNAs) targeting GCS1 and GRP78 were acquired from Tsingke, China, via U6-CMV-Puro vectors. And, flag-tagged GCS1 and its truncated variants were inserted into CMV-3Flag-PGK-Puro vectors. Myc-tagged GRP78 and His-tagged USP10 were inserted into pcDNA3.1 vector. Both the relevant negative control si-NC and the small interfering RNA (siRNA) targeting USP10 were acquired from Tsingke, China. As directed by the manufacturer, Lipofectamine™ 3000 reagents (Invitrogen, USA) were utilized for the transient transfection of plasmids into HEK293T cells. After two further days of growth, the transfected cells were chosen for one week with puromycin (Sigma-Aldrich). Western blotting or qRT-PCR was used to confirm the transfection effectiveness.

**RNA extraction and quantitative real-time PCR (RT‒qPCR) detection**

Total RNA was isolated from CRC tissue and cell lines via TRIzol reagent (Vazyme, China) in accordance with the manufacturer's instructions. Using reverse transcription PCR (RT-PCR) and the HiScript® III RT SuperMix (Vazyme, China), the isolated total RNA was reverse transcribed into cDNA. The genes were amplified and the expression levels of each gene were ascertained via quantitative real-time PCR (RT‒qPCR) using the ChamQ SYBR qPCR Master Mix (Vazyme, China). The findings were normalized to the GAPDH expression levels.

**Western blot Assay**

After total protein from CRC cell lines or tissues was extracted via RIPA lysis buffer (Beyotime, China), the protein concentration was determined via the bicinchoninic acid assay. The proteins were separated via a 10% SDS-polyacrylamide gel from Bio-Rad (America) and subsequently transferred to a Millipore polyvinylidene fluoride (PVDF) membrane. Afterward, the membrane was blocked for 30 minutes using QuickBlock™ blocking buffer (Beyotime, China). After being incubated overnight at 4°C, the membranes were washed three times for fifteen minutes each with TBST. Following another round of TBST, the PVDF membrane was placed into the secondary antibody solution and incubated at room temperature for two hours. Proteins were detected via an Omni-ECL™ Femto Light Chemiluminescence Kit (Epizyme Biotech, China). β-ACTIN was used as an internal control. The results were quantified by ImageJ software.

**Immunofluorescence staining**

The cells used in the experiment were seeded onto confocal microplates, allowed to grow overnight, and then fixed the next day via an immunostaining fixing solution. After that, the primary antibody solution was added to the small confocal dishes, which were then incubated overnight. After being cleaned, the fluorescent secondary antibody solution was added to the small confocal plates, which were then incubated at room temperature for two hours in the presence of a dark light source. The nuclei were stained with DAPI. Afterward, Leica SP5 confocal microscopy apparatus (Leica Microsystems, Germany) was used for observation, and preservation-related images were taken.

**Cell Proliferation Assays**

Trypsin was used to count and digest the CRC cells once they reached the logarithmic growth stage. There were several replicate wells in the 96-well plate, with 2 × 10^3^ cells in each well. The OD (450 nm) values were measured via a CCK-8 Cell Counting Kit (Beyotime, China) for four days. A second portion of the cells was inoculated into 6-well plates at 500 cells per well. These cells were then added to 3-well plates in duplicate. The cells were grown for 10–14 days, and the medium was completely changed every three days. Once the cell mass was visible to the unaided eye, it was stored with 4% paraformaldehyde. After that, it was photographed, stained with crystal violet, and counted via ImageJ software. After that, the remaining cells were cultured in DMEM supplemented with 10% fetal bovine serum for an entire day before EdU was added to a 96-well plate (1 × 10^4^ cells/well). In accordance with this technique, the cells were grown for two hours at 37°C, fixed for thirty minutes in 4% formaldehyde, and pierced for ten minutes at room temperature with 0.5% Triton X-100. After washing with PBS, 1 x azide 555 was added, and the mixture was allowed to react with EdU for 30 minutes. Next, Hoechst 33342 was added, and the reaction was observed for 10 minutes. A Nikon microscope was used to take pictures of the cells (Nikon, Japan).

**Invasion and migration assays**

The Transwell and wound healing assays were carried out in accordance with earlier reports^[1]^. Three arbitrary fields were chosen, and microscopy was used to measure them.

**Flow cytometric analysis of cell apoptosis**

The CRC cell apoptotic rate distribution was assessed via flow cytometry methods. The specific actions were carried out as previously described^[1]^.

**RNA‑sequence analysis**

HCT 116 vector-transformed cells and GCS1-overexpression cells were lysed with TRIzol (Vazyme, China). RNA extraction, library preparation, transcriptome sequencing and data analysis were performed by Personal Biotechnology (Shanghai, China).

**Supplementary reference:**

1. Shen, H., et al., *SCRIB Promotes Proliferation and Metastasis by Targeting Hippo/YAP Signalling in Colorectal Cancer.* Front Cell Dev Biol, 2021. **9**: p. 656359.

**Supplementary Figures:**


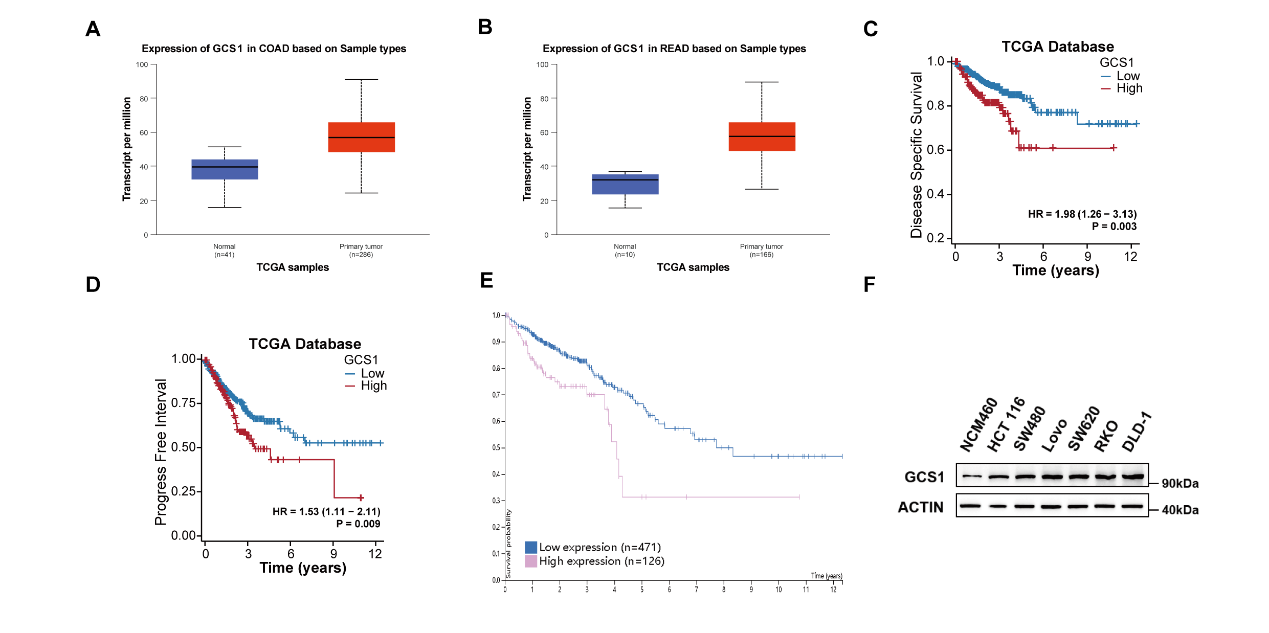


**Figure S1 High expression of GCS1 affects the survival of CRC patients.** **A, B:** Based on the UALCAN database, GCS1 expression was detected in both colon and rectal cancer. **C, D:** In the TCGA database, the associations between GCS1 score and disease-specific survival (DSS) and progression-free interval (PFI) were shown. **E:** In the HPA database, high expression of GCS1 predicted a shorter OS time. **F:** Expression of GCS1 at the protein level in normal intestinal epithelial cells and CRC cells.


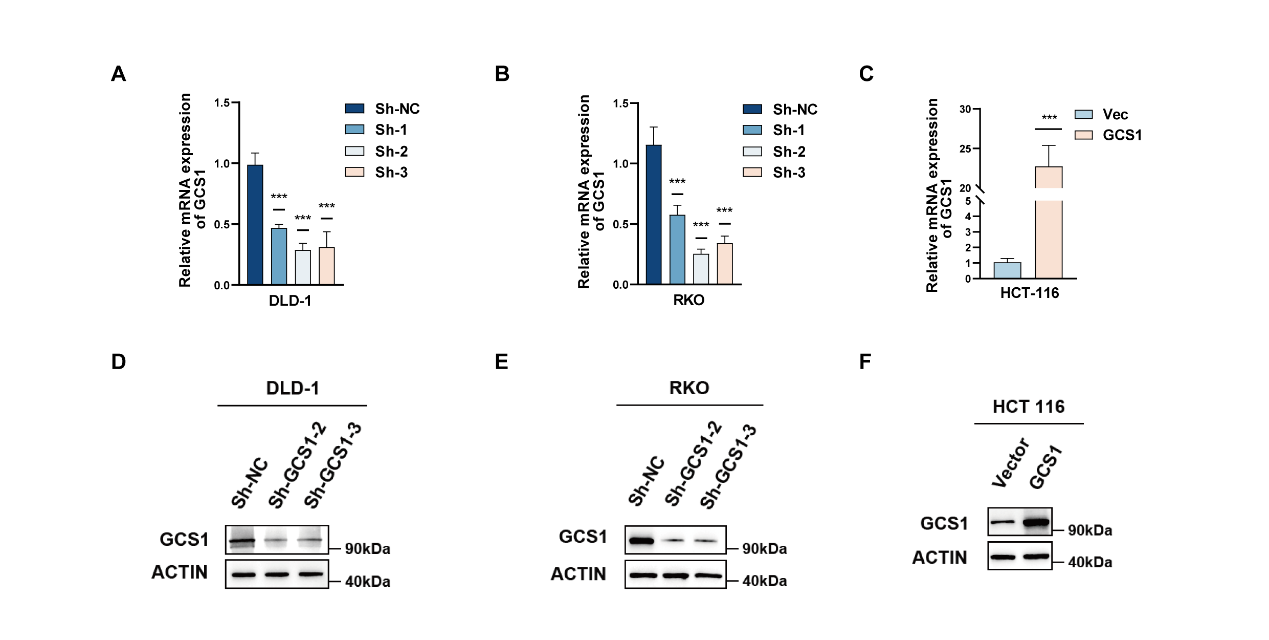


**Figure S2 Validation of the knockdown and overexpression efficiency of GCS1 in CRC cell lines. A, B:** RT‒qPCR was performed to confirm the knockdown efficiency of GCS1 in DLD-1 and RKO cells. **C:** RT‒qPCR was performed to confirm the GCS1 overexpression efficiency in HCT 116 cells. **D, E:** The protein level of GCS1 knockdown was confirmed in DLD-1 and RKO cells. **F:** Protein level verification was conducted to confirm the GCS1 overexpression efficiency in HCT 116 cells. The error bars indicate the mean ± SD of three independent experiments. *** P<0.001.


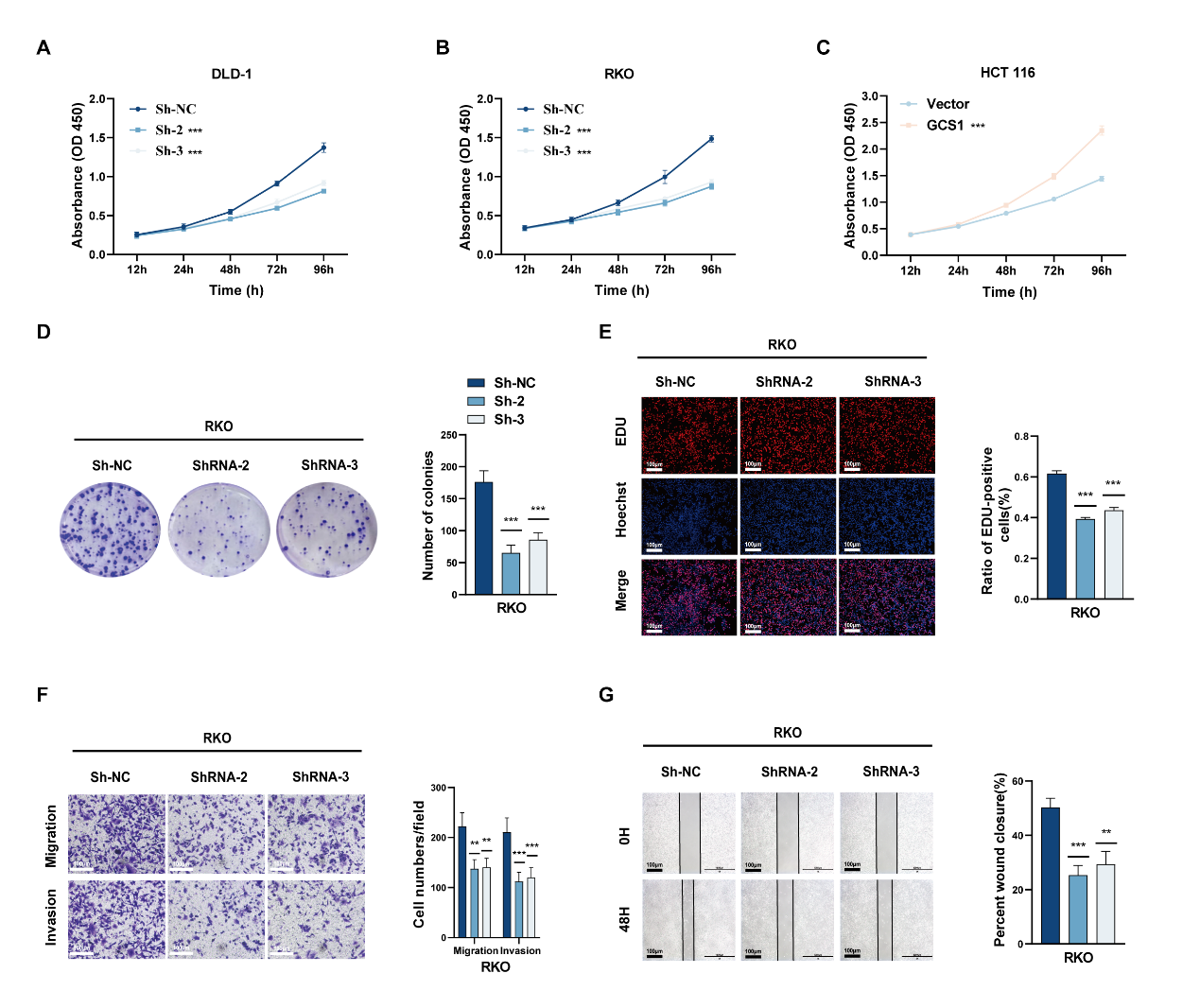


**Figure S3 GCS1 promotes the malignant phenotype of CRC in vitro. A-C:** CCK8 assay of HCT 116 cells with stable GCS1 overexpression and DLD-1 and RKO cells with stable GCS1 knockdown. **D:** Representative images and quantity of colonies informed by RKO cells with stable GCS1 knockdown. **E:** EdU-positive cell count and representative images of RKO cells with stable GCS1 knockdown. Scale bars, 100 μm. **F:** Representative images showing the number of migrating or invading RKO cells with stable GCS1 knockdown. Scale bars, 100 μm. **G:** Wound closure rate and representative images from the wound healing assay using RKO cells with stable knockdown of GCS1. Scale bars, 100 μm. The error bars indicate the mean ± SD of three independent experiments. ** P<0.01; *** P<0.001.


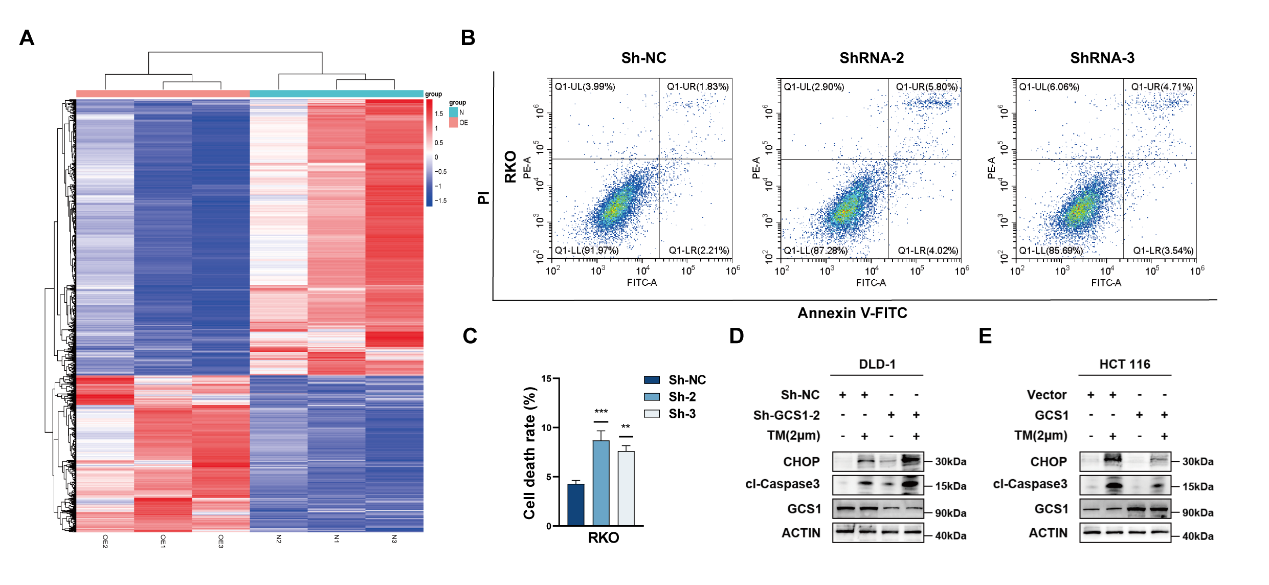


**Figure S4 GCS1 alleviates ER stress-mediated apoptosis.** **A:** Heatmap of the RNA sequencing results. **B:** Knockdown of GCS1 increased apoptosis in RKO cells. **C:** Statistical analysis of apoptosis in RKO cells. **D, E:** The expression of proapoptotic proteins was detected in cells in which GCS1 was knocked down or overexpressed after TM (2 μM) treatment. The error bars indicate the mean ± SD of three independent experiments. ** P<0.01; *** P<0.001.


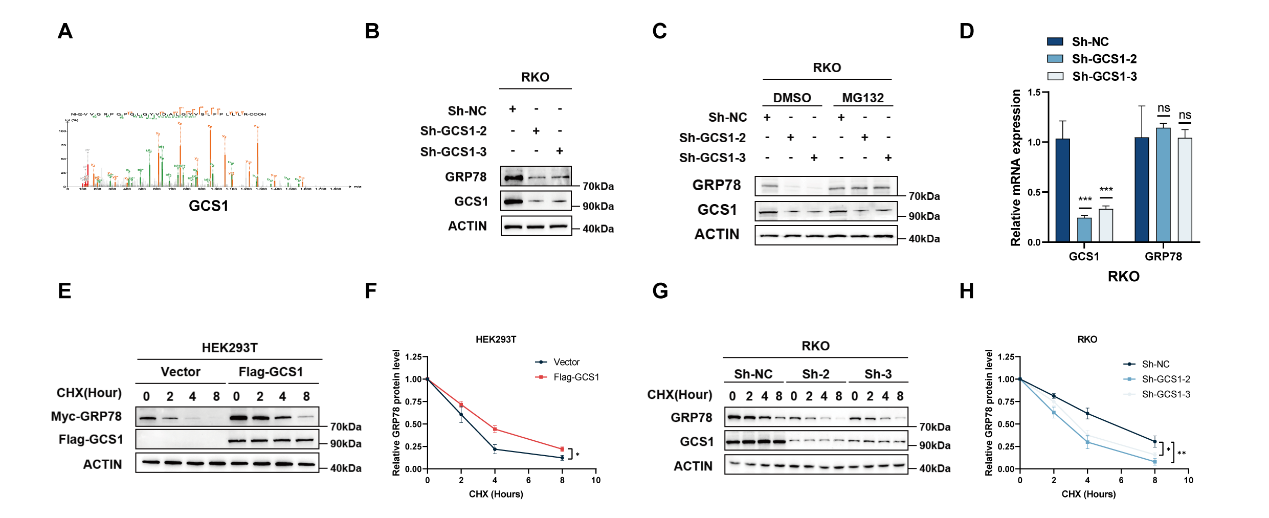


**Figure S5 GCS1 regulates the ubiquitination-mediated degradation of GRP78. A:** Specific peptides of GCS1 were identified. **B:** Reduced expression of GRP78 was observed in RKO cells with GCS1 knockdown. **C:** GRP78 expression was measured in RKO cells with GCS1 knockdown, following treatment with MG132 (10 μM). **D:** Changes in GRP78 expression following the knockdown or overexpression of GCS1 at the transcriptional level. **E-H:** GCS1 overexpression or knockdown in HEK293T and RKO cells, followed by CHX (100 μg/mL) treatment was used to measure alterations in GRP78 expression. The error bars indicate the mean ± SD of three independent experiments. ns, not significant, * P<0.05; ** P<0.01.


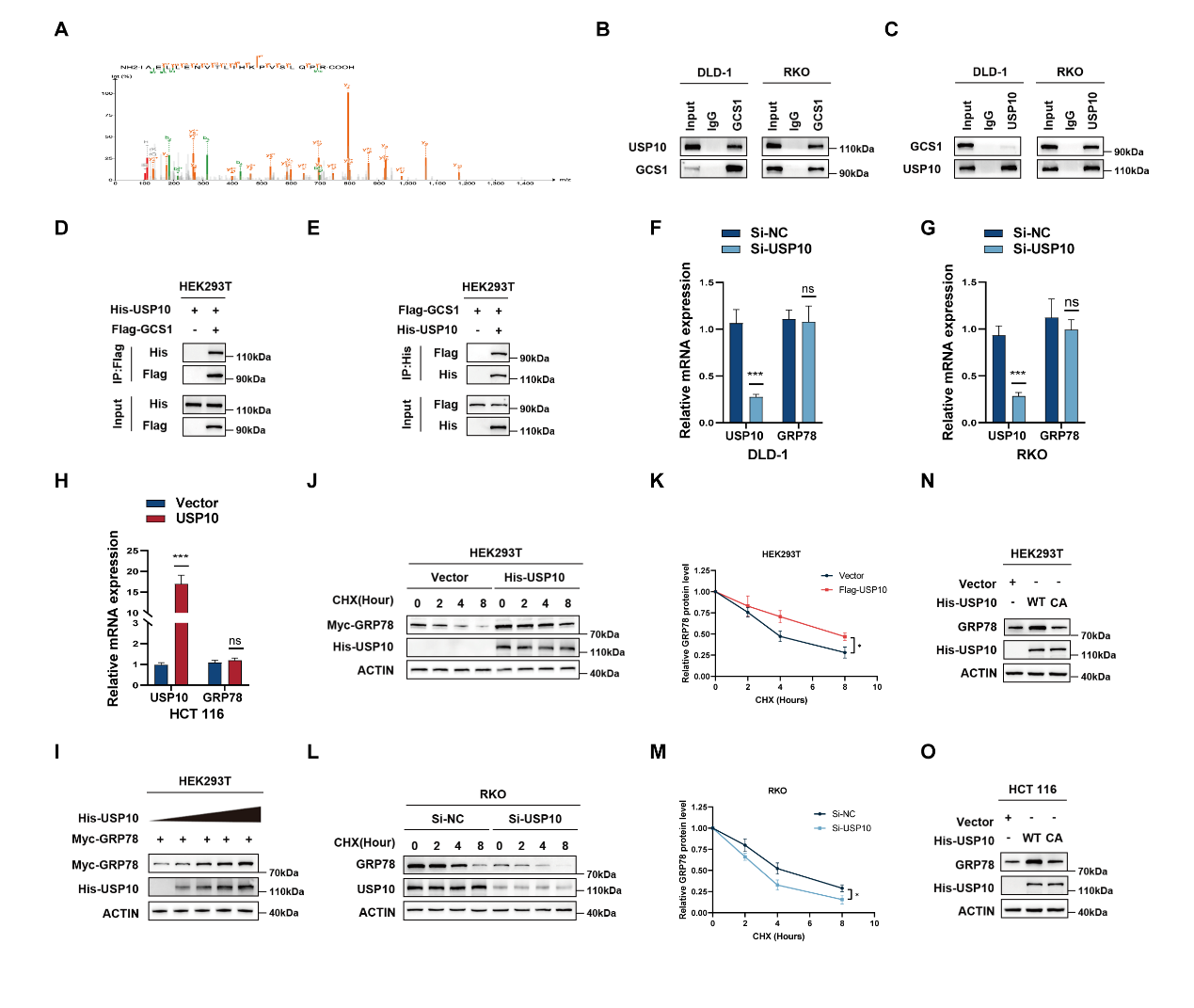


**Figure S6 GCS1 recruits USP10 to regulate the level of ubiquitinated GRP78. A:** Specific peptides of USP10. **B, C:** Endogenous validation of USP10 and GCS1 in combination. **D, E:** External confirmation of USP10 and GCS1 binding. **F–H:** Following USP10 overexpression or knockdown, alterations in GRP78 RNA expression were observed. I: To determine the expression of GRP78 in HEK293T cells, the concentration of USP10 plasmid was progressively increased. **J–M:** USP10 was overexpressed or knocked down in HEK293T and RKO cells, respectively, and the protein expression of GRP78 was detected after the cells were treated with CHX. **N, O:** USP10 (WT) and the USP10 mutant (C424A) were overexpressed in HEK293T and HCT 116 cells to detect the protein expression level of GRP78. The error bars indicate the mean ± SD of three independent experiments. * P<0.05.


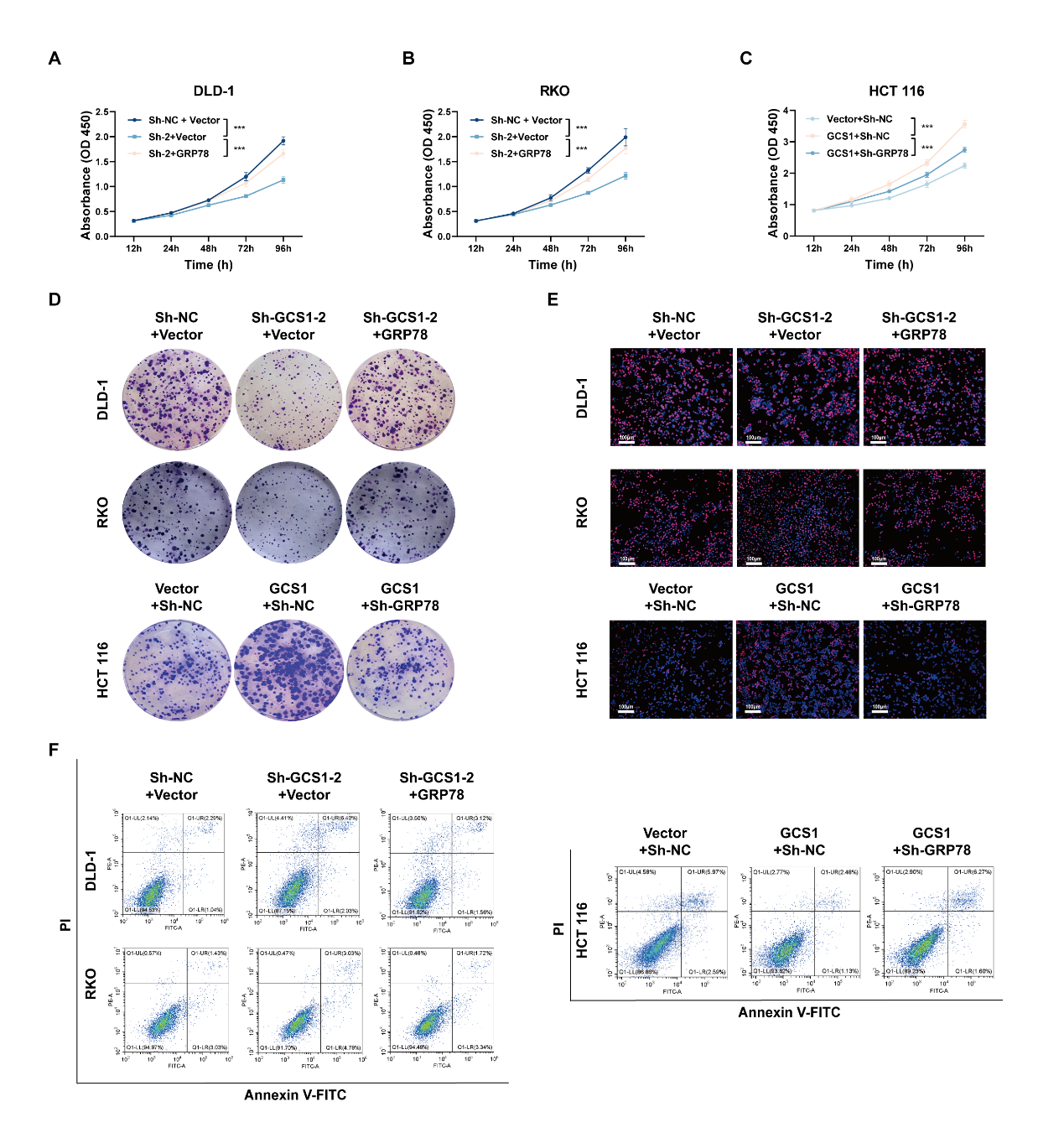


**Figure S7 GCS1 affects the proliferation and apoptosis of CRC cells regulated by GRP78. A-C:** The rescue effect of GRP78 overexpression on GCS1 knockdown cells or GRP78 silencing on GCS1-overexpressing cells was detected via a CCK-8 assay. **D, E:** Using colony formation and EDU assays, the rescue effect of the abovementioned three cell lines was identified. Scale bars, 100 μm. **F:** The rescue impact of the abovementioned three cell lines was determined via flow cytometric analysis. The error bars indicate the mean ± SD of three independent experiments. *** P<0.001.


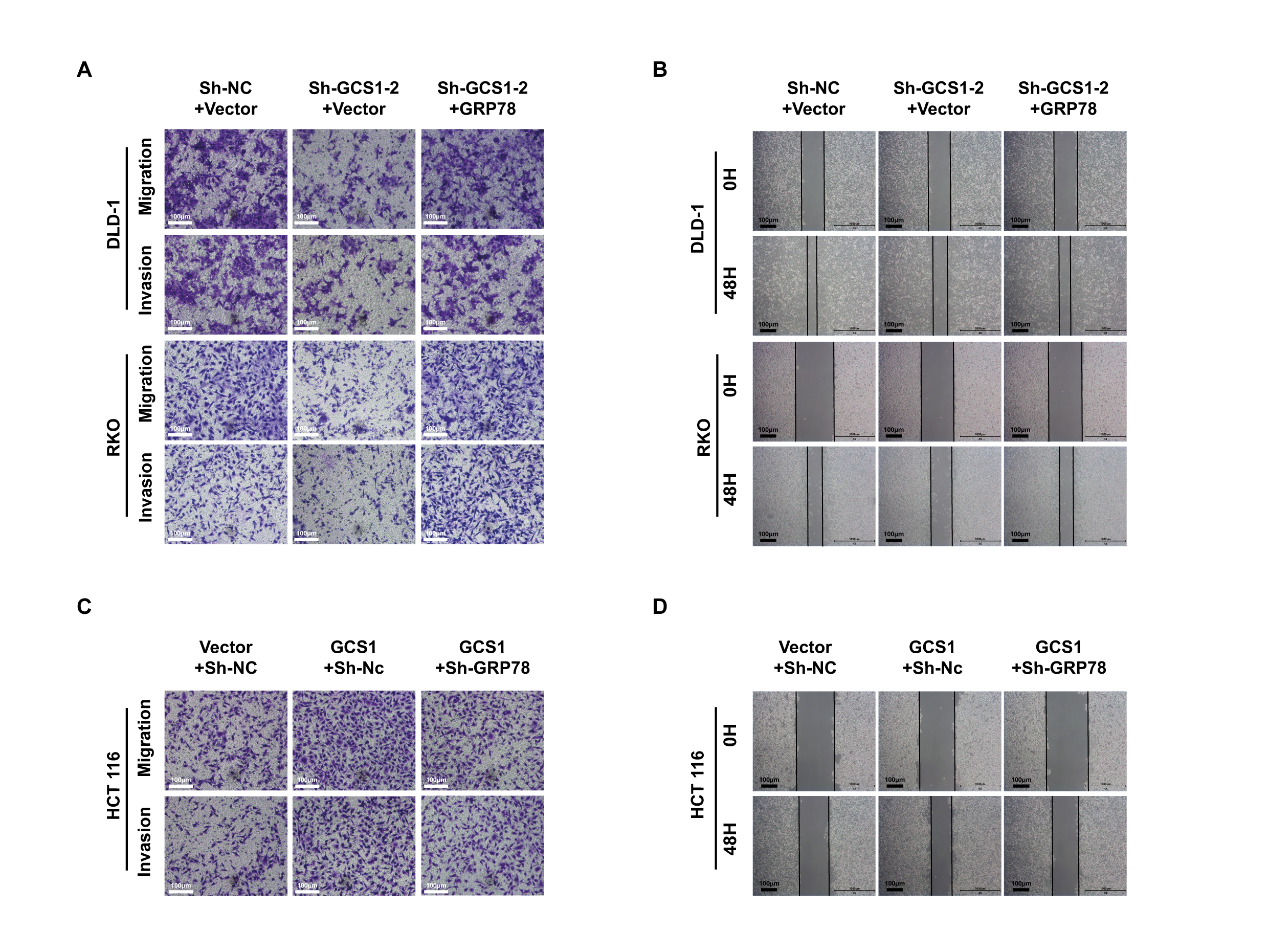


**Figure S8 GCS1 affects the migration and invasion of CRC cells via GRP78. A, B:** Transwell and wound healing assays were used to determine the rescue effect of GRP78 overexpression on GCS1-knockdown cells. Scale bars, 100 μm. **C, D:** The rescue effect of GRP78 silencing on GCS1-overexpression cells was determined via Transwell and wound healing assays. Scale bars, 100 μm.


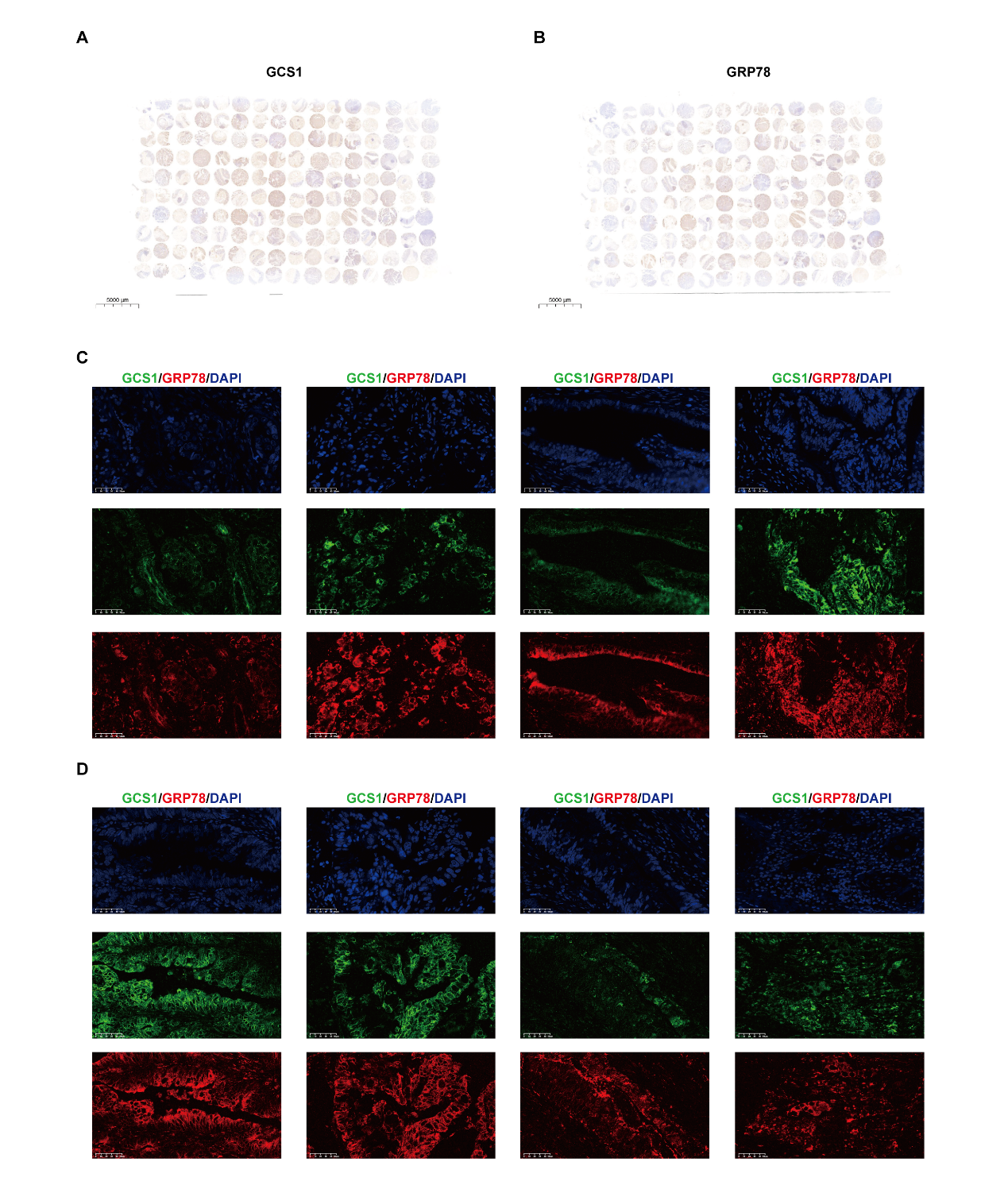


**Figure S9 IHC and IF results for GCS1 and GRP78 in the TMA. A, B:** IHC staining of GCS1 and GRP78 in the TMA (n = 80). **C, D:** Representative images of IF staining for GCS1 and GRP78 in tumor tissue from the TMA (n = 80). Scale bars, 50 μm.

**Supplementary Tables:**

**Table S1 Correlation between the GCS1 and clinicopathologic characteristics in patients from CRC.**

| **Clinicopathological**  **variables** | **Relative GCS1 Expression** | | **P value** |
| --- | --- | --- | --- |
|  | **Low (40)** | **High (40)** |  |
| **Gender**  Male  Female | 30  10 | 22  18 | 0.101 |
| **Age**  ≤60  > 60 | 10  30 | 16  24 | 0.233 |
| **T stage**  T1+T2  T3+T4 | 12  28 | 5  35 | 0.101 |
| **Lymph metastasis**  No  Yes | 25  15 | 13  27 | **0.027** |
| **Distant metastasis**  No  Yes | 37  3 | 36  4 | 0.710 |
| **Tumor size (cm)**  ≤5  >5 | 22  18 | 19  21 | 0.538 |
| **Vascular invasion**  No  Yes | 36  4 | 31  9 | 0.294 |
| **Nerve invasion**  No  Yes | 36  4 | 33  7 | 0.651 |
| **Tumor stage**  1+2  3+4 | 25  15 | 12  28 | **0.020** |
|  |  |  |  |

**Table S2 Primers used in this study.**

| For RT-qPCR | Primer sequence (5’→3’) |
| --- | --- |
| GCS1-Forward | AGTGACTGTAGAGCCTCAGGA |
| GCS1-Reverse | CCCACTGATAAACTTCAACTGCC |
| GRP78-Forward | GAAAGAAGGTTACCCATGCAGT |
| GRP78-Reverse | CAGGCCATAAGCAATAGCAGC |
| USP10-Forward | ATTGAGTTTGGTGTCGATGAAGT |
| USP10-Reverse | GGAGCCATAGCTTGCTTCTTTAG |
| GAPDH-Forward | CCAGAACATCATCCCTGCCT |
| GAPDH-Reverse | CCTGCTTCACCACCTTCTTG |

**Table S3 Antibodies used in this study.**

| Antibody | Source | Application |
| --- | --- | --- |
| GCS1 | Santa Cruz Biotechnology | WB/ IP/ IF/ IHC |
| GCS1 | Proteintech | WB |
| GRP78 | Proteintech | IF/ IHC/ IP/ WB |
| CHOP | Proteintech | WB/ IHC |
| cleaved-Caspase 3 | Cell Signaling Technology | WB/IHC |
| Ki-67 | Proteintech | IHC |
| Ubiquitin | Santa Cruz Biotechnology | IP/ WB |
| Myc | Proteintech | IP/ WB |
| DYKDDDDK(Flag) | Proteintech | IP/ WB |
| His | Proteintech | IP/ WB |
| HA | Proteintech | IP/ WB |
| goat anti-mouse IgG Cy3 | Invitrogen | IF |
| goat anti-rabbit IgG FITC | Invitrogen | IF |

**Table S4 Sh-RNAs and Si-RNA used in this study.**

| Gene | Sequence (5’→3’) |
| --- | --- |
| Sh-GCS1-1 | GAGGUATAGAGTTGCCAGCT |
| Sh-GCS1-2 | GGCAGTTCTTGATACAGCA |
| Sh-GCS1-3 | GCAGTATGTAGATGZCTCTT |
| Sh-GRP78 | GAAATCGAAAGGATGGTTAAT |
| Si-USP10 | CGACAAGCUCUUGGAGAUAAATT |
